# Impact of DNA Extraction Strategies on Genomic and Bioinformatic Outcomes in Eight Selected Fungal Strains

**DOI:** 10.3390/jof12050299

**Published:** 2026-04-22

**Authors:** Cyrine Abid, Hela Zouari-Mechichi, Riadh Benmarzoug, Tahar Mechichi, Najla Kharrat

**Affiliations:** 1 National School of Engineers of Sfax, University of Sfax, Soukra Road Km 4, P.O. Box 1173, Sfax 3038, Tunisia; 2 Laboratory of Molecular and Cellular Screening Process, Centre of Biotechnology of Sfax, University of Sfax, Sidi Mansour Road Km 6, P.O. Box 1177, Sfax 3018, Tunisia; riadh.benmarzoug@cbs.rnrt.tn (R.B.); najla.kharrat@cbs.rnrt.tn (N.K.); 3 Laboratory of Biochemistry and Enzymatic Engineering of Lipases, National School of Engineers of Sfax, University of Sfax, Soukra Road Km 4, P.O. Box 1173, Sfax 3038, Tunisia; hela.zouari@isbs.usf.tn (H.Z.-M.); tahar.mechichi@enis.rnu.tn (T.M.); 4 Institute of Biotechnology of Sfax, University of Sfax, Soukra Road Km 4, P.O. Box 1175, Sfax 3038, Tunisia

**Keywords:** bioinformatics, Illumina, mycelium, nanopore, PCR amplification

## Abstract

High-quality genomic DNA extraction remains a major bottleneck for fungal genomics, particularly for worldwide aerobic and non-photosynthetic mushroom species that rely on their rigid cell walls, interference between metabolites, polysaccharides, etc., and complex genomes. This study systematically compares five DNA extraction protocols involving four distinct sample preparation procedures (fresh (A), filtered (B), frozen (C) and cryogenic mycelium (D)) across mycelial cultures of eight Tunisian fungal strains representing *Ascomycota* and *Basidiomycota* to identify the optimal combination for genomic DNA extraction from mycelium. The eight phylogenetically diverse fungal species were analyzed using short-read (MiSeq and NextSeq550) and/or long-read (MinION Mk1C) sequencing technologies, giving a depth coverage between 3.7× and 83×. The generation and quality of the assemblies were assessed within the Galaxy platform, which revealed a gap percentage of 0–0.509%. Taxonomic characterization and phylogenetic inference were performed with SANGER technology using the Internal Transcribed Spacer (ITS) and D1/D2 region of the 26S rRNA gene, assigning the species to our eight different strains: *Clitopilus baronii* (BS6), *Porostereum spadiceum* (BS200), *Trametes versicolor* (BS22-9), *Schizophyllum commune* (BS23-13), *Gloeophyllum abietinum* (BS23-14), *Irpex laceratus* (BS100), *Trichoderma asperellum* (GC9) and *Trichoderma harzianum* (S3). The optimized DNeasy Plant Pro Kit protocol with cryogenic biomass treatment presents a safe and cost-effective method for fungal genome sequencing and taxonomic resolution. This integrated comparative evaluation of extraction for sequencing identifies an optimal Qiagen-based extraction strategy combined with cryogenic treatment for eight diverse Tunisian fungal species, guiding method selection based on specific cell wall characteristics rather than proposing a universal protocol limited by unequal replication and strain numbers.

## 1. Introduction

During their lifecycle, fungi go through two main phases: vegetative growth (mycelium) and reproduction [1]. Mycelia can proliferate in distinct environments depending on substrates, fungal type, and growth conditions [2,3]. This ability is facilitated by the synthesis of various active biological compounds with potential therapeutic functions, identified as proteins or/and primary and secondary metabolites (glutathione, ergothioneine, etc.) [4].

Multicellular filamentous molds, made of very thin threads known as hyphae (2–10 μm), grow and keep interconnecting, creating a network structure called mycelium (centimeter- to meter-scale). The growth of macroscopic filamentous fungi is also based on underground-developed mycelium. However, macrofungal strains differ by producing observable sporocarps at the surface, called fruiting bodies, commonly known as mushrooms or toadstools [4,5].

Among the estimated number (3–6 million) of non-photosynthetic and aerobic mushroom species, only a small fraction, 3–8%, have been accurately and precisely identified, i.e., about 200,000 species [3,6,7,8,9]. Indeed, mushrooms’ worldwide diversity has not yet been mapped, identified and categorized, even after the increasing interest in their unexploitable potential due to limited distinctive morphological characteristics and unsuitable microscopic features, especially due to the challenging taxonomic identification of *Basidiomycota* phylum species [3,7,10,11]. Modern classifications based on rDNA sequences have suggested that the fungi kingdom is composed by four major phyla, namely *Zygomycota*, *Chytridiomycota*, *Basidiomycota*, and *Ascomycota*, although Wijayawardene et al., 2024, proposed 19 distinct lineages [2,12].

In recent years, OMICs studies of mushrooms have grown considerably and have addressed the characterization of *Ascomycota* (average genome size of 37 Mb) and mainly *Basidiomycota* species (average genome size of 46 Mb). In fact, extracting genomic DNA (gDNA), especially from both phyla, is highly challenging due to their large and complex genome architecture, robust cell walls, high polysaccharide content, and secondary metabolites. To follow this evolving interest, many protocols and technological improvements in short- and long-read sequencing technologies have been developed [11,13,14].

In fact, the quality of gDNA sequence reads depends on the quality and the quantity of the extracted gDNA. Extracting gDNA from tissue or mycelium depends on efficient cell lysis, which itself depends on mechanical, physical–mechanical and enzymatic techniques (grinding in liquid nitrogen, disruption with glass beads, cell wall digesting enzymes like chitinase and cellulase, etc.). Fungi are known to be rich in various substances, such as polysaccharides and polyphenols, which are difficult to remove from DNA extracts, in addition to proteins and RNA. While universal methods and many commercial kits and several home-brew methods are available to extract gDNA from mushroom tissues (cap/pilei and/or stalk/stems) and/or mycelium, their respective efficiencies may vary across fungal strains due to the wide and diverse macrofungal characteristics (morphology, metabolites, cell rigidity and composition, co-precipitating contaminants, PCR inhibitors, etc.) [15,16,17].

Although commercial kits are cost-effective and time-efficient, distinguishing between commercial kits and home-brew methods is quite challenging and requires a comparative evaluation of extraction strategies, especially if researchers have to compromise between the gDNA purity and maximizing its yield for taxonomic characterization and high-throughput sequencing.

This study aims to optimize a selected protocol, specific to the matrix and phylogeny, to maximize the yield, purity and integrity of genomic DNA. To this end, a systematic comparison of five different extraction protocols, associated with four types of sample preparation, was applied to the mycelium of eight Tunisian fungal species, exhibiting underrepresented biodiversity. Our work evaluates method efficiency—the most appropriate combination of extraction method and sample preparation—particularly for Internal Transcribed Spacer (ITS) and D1/D2 genomic regions, as well as for short- and/or long-read sequencing technologies, rather than proposing a universal standardized strategy across fungal species/strains.

## 2. Materials and Methods

All pipette tips, tubes and solutions were autoclaved to avoid non-target gDNA contamination. All chemicals used in this study were of analytical grade. For gDNA extractions, the chloroform:isoamyl alcohol (24:1, *v*/*v*) was obtained from Thermo Fisher Scientific (Waltham, MA, USA), ethanol absolute (≥99.8%) from Novachim (Marseille, France) and the phenol:chloroform:isoamyl alcohol (25:24:1, *v*/*v*/*v*) from PanReac (Castellar del Vallès, Spain). All applied protocols from extraction to library preparation were performed at room temperature.

### 2.1. Fungi Mycelia Strain, Growth, Culture Condition and Storage

**Figure 1:**
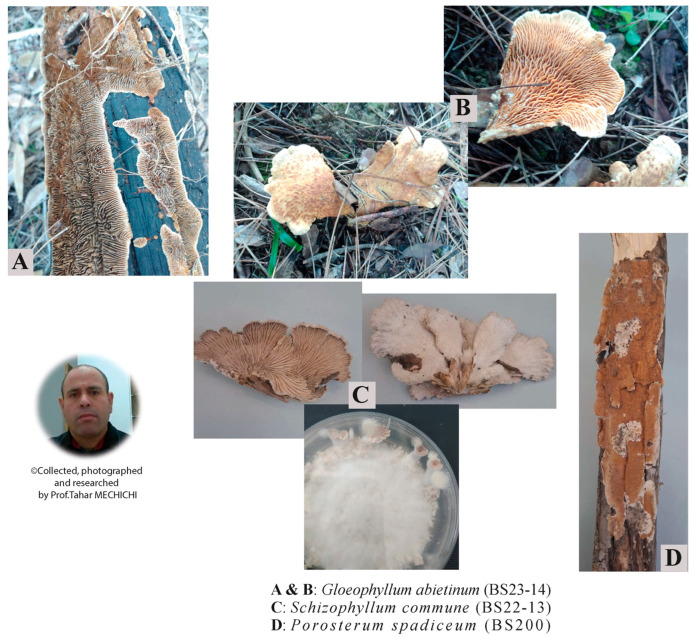
Some of the selected Tunisian macrofungal species.

The gDNA was extracted from different types of pure mycelium Tunisian fungi samples in a private collection, from eight species. These strains, belonging to the *Ascomycota* and *Basidiomycota* phyla, were collected from different habitats in the Northwest Region (Ain Drahem, Bousalem, and Kef) of Tunisia and were obtained as pure mycelia cultures maintained on Potato Dextrose Agar (PDA) medium (Figure 1 and Table 1). The selected panel reflects typical laboratory use rather than exhaustive taxonomic coverage.

**Figure 2:**
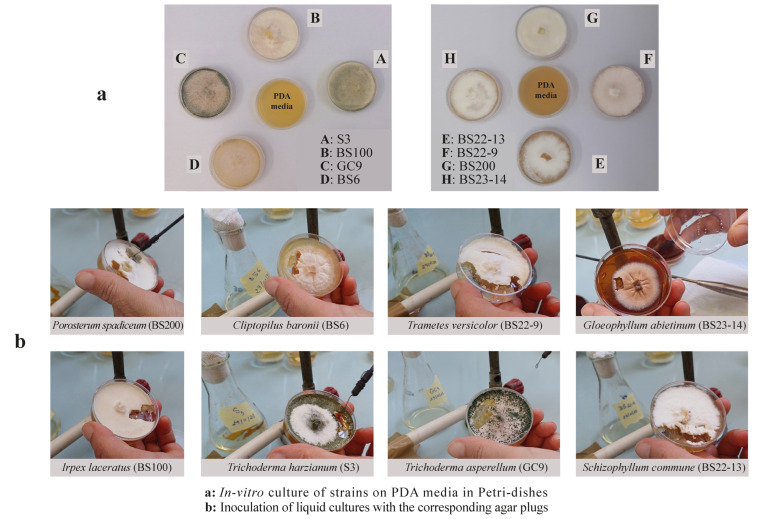
The eight pure Tunisian strains in their preserved vegetative growth phase.

Strains were grown on PDA media in Petri dishes (Figure 2a). Then liquid cultures were prepared in 50 mL Erlenmeyer flasks containing 25 mL of M7 medium at pH 5.5. M7 medium was prepared as described in Zouari-Mechichi et al. (2006) without any induction for laccase activity and was sterilized by autoclaving at 120 °C for 30 min [18]. Each liquid culture was inoculated with the corresponding agar plugs (≈0.3 cm^2^) cut from the growing edge of the freshly plated solid fungal culture and then incubated at 25–30 °C without stirring for 7–10 days until fungal growth was observed (Figure 2b). This regional collection highlights the extraction challenges related to the cell wall chemistry specific to each strain, offering insights applicable to non-model fungi worldwide.

### 2.2. Types of Sample Preparation

*Porostereum spadiceum* (strain BS200) pure mycelium was randomly selected as the reference strain for comparison between protocols. Simplicity, efficiency, profitability, repeatability and reproducibility were the criteria for selecting the best sample preparation method (see Figure S1).

Four different sample preparation methods were used (denoted A, B, C, and D). Preparation A was obtained by collecting the fresh, non-filtered and sterile-grown mycelium for immediate use (see Figure S1a). Figure S1c shows the non-sterile filtering system used to separate and collect the vegetative mycelium from the culture medium (preparation B). Preparations C and D were based on sterile freshly collected mycelium stored at −20 °C for a minimum of 24 h. On a sterile medical compress, every sample, after thawing at room temperature, was rinsed with autoclaved ultrapure water (≈3 mL) and then manually squeezed to obtain preparation C (see Figure S1b from A to B). The pre-D sample was treated the same as C, on the same day with the same experimental conditions, and then treated with liquid nitrogen by grinding the samples into fine powder by mechanical lysis to obtain preparation D (mortar and pestle) (see Figure S1b from A to C). All mycelium samples treated with liquid nitrogen were directly put in the lysis tube (included in the selected kit) and stored at −20 °C for 48 h before gDNA extraction.

### 2.3. Genomic DNA Extraction Methods

**Figure 3:**
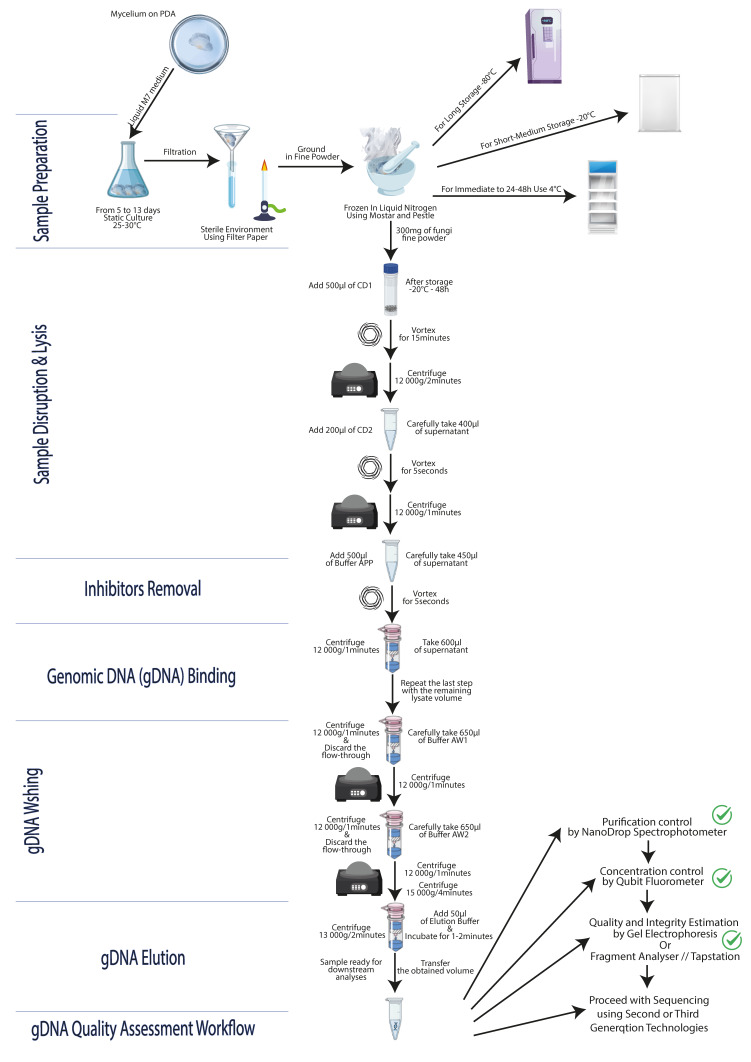
Schematic representation of the workflow of the optimized genomic DNA extraction protocol.

To compare the performance of 10 applied extraction protocols on different fungal species belonging to the *Basidiomycota* and *Ascomycota* phyla, five different methods were tested, including the phenol–chloroform extraction protocol [19] and four commercial kits (see Table S1 and Table 2 and Figure 3). Due to constraints in sample material and reagent availability, replications per protocol were unbalanced, therefore limiting any formal statistical capability for comprehensive comparisons among all methods.

### 2.4. Molecular Identification of Selected Strains

The non-coding Internal Transcribed Spacer (ITS) and the divergent coding domains (D1/D2) of ribosomal DNA were sequenced for taxonomy annotation of the fungal strains [20].

#### 2.4.1. PCR Amplification

To evaluate the quality of extracted gDNA, Polymerase Chain Reaction (PCR) was employed to amplify the ITS region and D1/D2 domain, with negative controls ensuring the PCR amplicons’ purity and three independent replicate reactions per sample. The ITS region was amplified using the flanking primers ITS1 and ITS4, in the 18S and the 28S subunit to amplify a unique band of ITSI, ITSII and 5.8Sr. The hypervariable D1/D2 domain, part of the nuclear Large SubUnit (LSU) 28S ribosomal RNA, was amplified with NL1 and NL4 primers [21].

**Figure 4:**
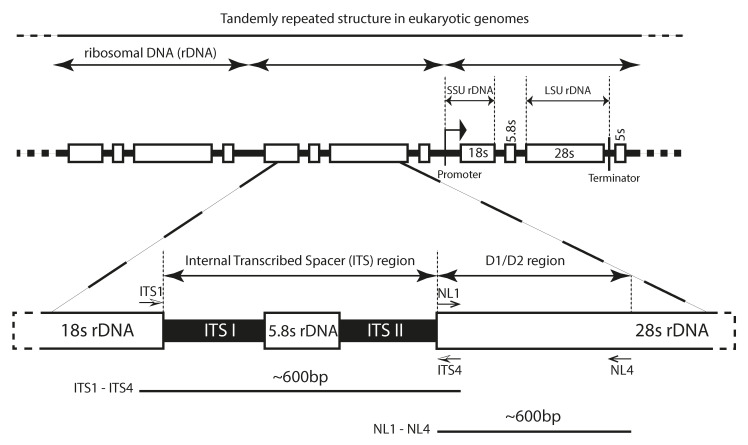
Schematic representation of the fungal ribosomal genes for the ITS regions and D1/D2 domain amplified in this study.

PCR amplification were performed with each pair—ITS1 (25 mM, CGAT_aligo 181105-116) and ITS4 (25 mM, CGAT_aligo 181105-121) (5′-TCCGTAGGTGAACCTGCGG-3′ and 5′-TCCTCCGCTTATTGATATGC-3′), and NL1 (576157—Q2582 (E06), 25 µg/OD, Invitrogen) and NL4 (576157—Q2582 (E05), 29 µg/OD, Invitrogen) (5′-GCATATCAATAAGCGGAGGAAAAG-3′ and 5′-GGTCCGTGTTTCAAGACGG-3′)—and taxonomical annotations were based on the reverse–forward sanger sequencing of the ITS region and D1/D2 domain, respectively (Figure 4).

A total of 0.2 µL of Q5 DNA polymerase (Q5^®^ High-Fidelity DNA Polymerase, M0491S, New England BioLabs, Ipswich, MA, USA) and 10 µL of Q5 buffer (5×) was mixed with forward (10 µM) and reverse (10 µM) primers (1 µL of each), 10 mM dNTP Mix (ThermoFisher Scientific, USA), and 1-2 µL of extracted gDNA (50 ng/µL) and completed with AmbionTM Free-Nuclease Water (AM9937, Invitrogen by ThermoFisher Scientific, USA) for a final volume of 50 µL.

PCR was conducted with an initial denaturation at 94 °C for 5 min followed by 35 cycles at 94 °C for 30 s, 58 °C (ITS1/ITS4) or 60 °C (NL1/NL2) for 30 s, and 72 °C for 30 s, with a final extension at 72 °C for 7 min, in a Thermal Cycler (MiniAmpTM, Applied Biosystems, Waltham, MA, USA). All PCR products (5 µL, undiluted) were migrated on a 2% agarose (cat#: EZEE-AG500, Cleaver Scientific, Rugby, UK) gel containing 2.5 µL/100 mL of Xpert Green DNA Stain (GS01.0001, GRISP Research Solutions, Porto, Portugal) and visualized using a UV transilluminator with a Bio-Print Gel Documentation Imaging System (Vilber, Collégien, France). The selected samples were sequenced with SeqStudioTM Flex Series Genetic Analyzers (Applied Biosystems, ThermoFisher Scientific, USA) after Exonuclease I (M0293S, New England BioLabs) treatment, according to the manufacturer’s instructions. Each sample for each region was amplified and sequenced at least two times.

#### 2.4.2. Phylogenetic Analysis and Taxonomic Annotation

Forward and reverse sequences of each region/domain were aligned with Bioedit Sequence Alignment Editor version 7.2 (https://bioedit.software.informer.com/7.2/, last access 14 February 2025), optimized and corrected manually and based on three replicates, when necessary, to obtain the consensus sequences.

A local alignment was performed using BLASTN (https://blast.ncbi.nlm.nih.gov/Blast.cgi?PROGRAM=blastn&PAGE_TYPE=BlastSearch&LINK_LOC=blasthome, last access 14 February 2025), a National Center for Biotechnology and Information (NCBI) open-source online tool, for ITS region and D1/D2 domain fungal strain identification against the Core nucleotide database (core_nt) by searching for Highly Similar Sequences (megablast) using the obtained consensus sequences as a query.

After running the BLAST (https://blast.ncbi.nlm.nih.gov/Blast.cgi, last access 14 February 2025 and selecting the strain sequences, a FASTA file was downloaded and aligned with the newly generated sequences using Molecular Evolutionary Genetics Analysis open-source software (MEGA, version 12, 64-bits, https://www.megasoftware.net/, last access 21 April 2025) to build the phylogenetic tree [22].

The MUSCLE algorithm was used to align the selected sequences using the default parameters, for better average accuracy and speed. The phylogenetic tree was generated using the Neighbor-Joining algorithm with the default distance (p-distance), default substitutional model (Maximum Composite Likelihood) and 1000 replicates for Slow Standard Bootstrap as bootstrap analyses to assess tree robustness [23]. The maximum likelihood method was used for combined sequences of ITS1, ITS2 and D1/D2 regions when applied to species identification with the *Tamura-Nei* model as the default substitutional model [24]. *Neurospora crassa*, *Lentinula edodes*, *Pleurotus ostreatus* and *Agaricus bisporus* were chosen as outgroups because they are the most known and identified macrofungal species [25,26,27,28]. ITS and D1/D2 sequences were uploaded into NCBI and their GenBank accession numbers are given in Table S2.

### 2.5. Genomic DNA Assessment Workflow

After gDNA extraction according to the above-described protocols (see Table S1), each sample was evaluated prior to downstream analyses with two different optical methods (NanoDrop and Qubit) and with electrophoresis gel.

#### 2.5.1. gDNA Purity Using NanoDrop Spectrophotometer

A clean and calibrated NanoDrop^®^-2000 instrument (Thermo Fisher Scientific, USA) was used to estimate DNA concentration (ng/μL) and sample purity based on the A260/A280 (≈1.8–2.0) and A260/A230 (≈2.0–2.2) absorption ratios. Each collected gDNA sample was tested using 1 μL after adjusting the appropriate blank (Elution Buffer specific to each kit, except for kit1, where autoclaved ultrapure H_2_O was used).

#### 2.5.2. gDNA Integrity Quality Estimation by Gel Electrophoresis

The presence, quality and gDNA integrity were then assessed by electrophoresis on 2% agarose gel (LE Agarose Multi-Purpose Agarose, Cleaver Scientific) using 1× TBE (Tris Borate EDTA) (0.1 M Tris, 0.09 M boric acid, and 1 mM EDTA) running buffer for 1 h at 100 V in a gel electrophoresis system (ENDUROTM) with a PowerPacTM Basic Power Supply (BIO-RAD, Hercules, CA, USA) and then visualized under UV light, with a Bio-Print Gel Documentation Imaging System (Vilber, France). The ethidium bromide (10 mg/mL, BIOTECH, Budapest, Hungary) was added to the agarose solution before electrophoresis. The gDNA volume (1 μL) was stained with a loading buffer (10× DreamTaq, Green Buffer, ThermoScientific) to monitor the progress of electrophoresis. The size estimation was based on the use of GeneON 100 bp Plus Blue DNA ladder (GeneON, cat.no. 304-105) and/or GeneON 1kb DNA ladder BLUE (GeneON, cat.no. 305-105).

#### 2.5.3. gDNA Quantification Assurance Using Qubit 3.0 Fluorometer

All collected gDNA concentrations were measured using a 1 μL sample with a Qubit3.0 Fluorometer (Invitrogen) using the Invitrogen QubitTM dsDNA BR Assay Kit (ThermoFisher Scientific) or/and Invitrogen QubitTM dsDNA HS Assay Kit (ThermoFisher Scientific), according to the manufacturer’s instructions.

The total gDNA yield was then estimated as follows:

Total gDNA yield (μg) = DNA concentration (μg/μL) × total DNA sample volume (μL)

### 2.6. Genomic DNA Sequencing Strategy

After gDNA assessment, the selected samples were sequenced using different libraries and Next-Generation Sequencing (NGS) platforms (see Table S3).

#### 2.6.1. Nanopore Oxford Technologies (ONT) Library Preparation, Sequencing and Base Calling

The ONT sequencing library was prepared using the Rapid Barcoding Sequencing Kit V14 (SQK-RBK114.24), following the manufacturer’s instructions (Oxford Nanopore Technologies, Oxford, UK). BS6 was sequenced twice. Both sequences were run on MinION Mk1C flow cell R10.4.1 under the control of MinKNOW 24.02.16 (Firmware MinION FPGA 2.4.3) with a difference in running time (72 h and 17 h). The base calling and filtering were done in real time and in an integrated way through the FAST base calling model, with eight as a minimum qscore and with ONT predefined parameters on MinION Mk1C to generate FastQ files.

#### 2.6.2. Illumina Library Preparation, Devices and Sequencing

The BS23-14 isolate was first prepared with the Nextera XT Kit and sequenced on an Illumina MiSeq platform in the 2 × 250 bp paired-end read mode with kit MiSeq v2 (insert size equal to 500 bp). Libraries for the eight gDNA Tunisian strains were then prepared with the Illumina DNA Prep kit to generate 2 × 150 bp paired-end reads with a 300 bp insert size and sequenced on an Illumina NextSeq550 platform with a mid-output Nextseq550 kit and 300 cycles (with 3 other samples not mentioned in this work). All Illumina libraries were prepared using official Illumina kits and according to the standard manufacturer’s protocols (Illumina Technology, San Diego, CA, USA).

### 2.7. Quality of Generated Raw Data and Bioinformatic Analyses

The quality of the raw Illumina and Nanopore sequence reads was assessed with FastQC (version 0.11.9) and then MultiQC (v1.0) using the line command Ubuntu22, launched with default parameters. To obtain all drafted assemblies, raw data were analyzed using a locally installed Galaxy server (v23.1, Galaxy 2023), through the following steps: (1) trim the raw data with Trimmomatic v.0.39 or Porechop v.0.2.4; (2) assemble with SPAdes v.3.15.4 or Flye Assembler v2.9.1 for Illumina or Nanopore reads, respectively; (3) complete assembly quality check with two different tools of the assemblies (QUAST v.5.2.0 and gfastats v.1.3.6); (4) check the presence of conserved and single-copy genes using BUSCO v.5.4.6 (see Table S4).

### 2.8. Nucleotide Sequence and Whole Genome Sequencing (WGS) Accession Numbers

The data reported in this work for the nucleotide sequence of the ITS region and D1/D2 domain and Whole Genome Sequencing project of the eight different Tunisian fungal strains have been deposited in the DDBJ/ENA/GenBank databases under the accession numbers listed in Table S5 and under the BioProject PRJNA1215380 (https://www.ncbi.nlm.nih.gov/bioproject/PRJNA1215380, last access 17 August 2025).

## 3. Results

### 3.1. Fungi Strains and Experimental Design Strategy

A pure culture of each studied strain (BS6, BS200, BS22-9, BS23-13, BS23-14, BS100, and S3) was obtained after the growth of mycelia from fungal crust collected on decayed wood, except for GC9, which was isolated from a chemical industry effluent (Table 1). BS200, BS6 and BS23-14 required 16 days of culture, while the other strains needed only nine days to grow, except BS22-13, which required 13 days. GC9 and S3 belong to the largest phylum of the fungi *Ascomycota* and present a green pigmentation, but the remaining six strains are *Basidiomycota* (Figure 2).

Because replication was uneven across protocols, we did not perform formal statistical comparisons for all pairwise contrasts, and the reported differences should be interpreted descriptively. We therefore adopted a decision-making process based on subjective analysis. We used sterile fresh and filtered mycelia (preparation B), from the same culture of BS200, as the standard strain (randomly selected), to compare the extraction protocols. The phenol–chloroform extraction method (see Table 1, Protocol n°1) was considered by verifying the quality and the quantity of two previous gDNA extracts of two other mycelia (BS22-9 and BS23-14). Using a high quantity of mycelium yield, this method provides a good quantity with poor quality. This in-house method is a well-known practice for amplicon and metagenomics sequencing [29]. However, it has low throughput and high toxicity (hazardous reagents) and takes a long time. Between QIAamp DNA Microbiome (kit 2), Plant/Fungi DNA Isolation (kit 3), Genomic DNA Purification Plant (kit 4), and DNeasy Plant Pro (kit 5), kit 2 and kit 5 seem to be the more interesting (Table 2; see Table S6a; see Figure S2).

To improve the efficiency of QIAamp DNA Microbiome, the BS200 mycelia was treated with alumina. This did not improve yield, even with 0.5 g of treated mycelia compared to 0.2 g without alumina. However, with nearly a 0.5 g yield of BS200 or BS6, the optimized protocol of the QIAamp DNA Microbiome Kit gave a good quantity and quality of the gDNA extract (see Table S6a). The DNeasy Plant Pro Kit (kit 5), with the manufacturer’s instructions, showed higher performance in terms of both yield and gDNA quality (see Table S6c). On the other hand, we observed no difference between the gDNA extracts of BS200 with or without the PS solution provided with kit 5 (see Table S6b). Based on these results, a pre-optimized protocol was set for kit 5 (see Table S1—Protocol n°9).

**Figure 5:**
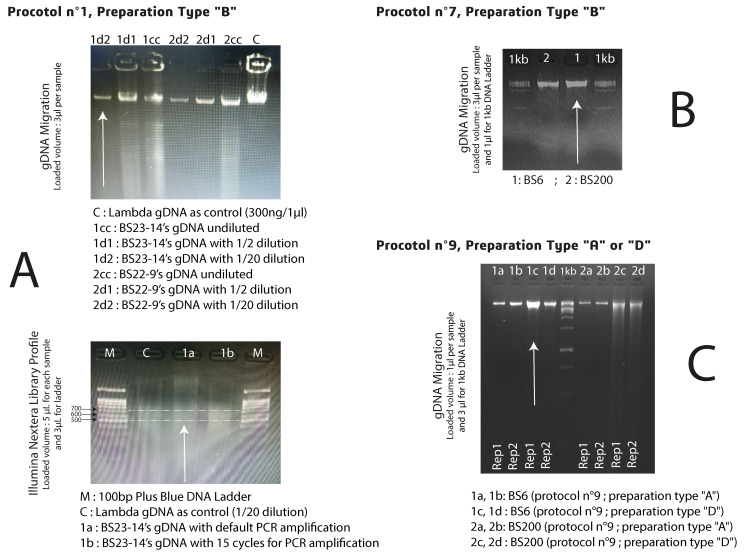
Agarose gel (2%, *w*/*v*) of gDNA obtained from BS6 and BS200 mycelium to compare between the different applied protocols. (**A**) gDNA extracts with Phenol-Chloroform method-upper section|MiSeq library profile-down section. The white arrow, in the upper section, indicate the selected gDNA sample for MiSeq platform; (**B**) gDNA extracts with kit5 manufacturer’s instructions. The white arrow indicate the selected gDNA sample for the first run of MinION Mk1C; (**C**) gDNA extracts with pre-optimized protocol. The white arrow indicate the selected gDNA sample used for the second run of MinION Mk1C.

**Figure 6:**
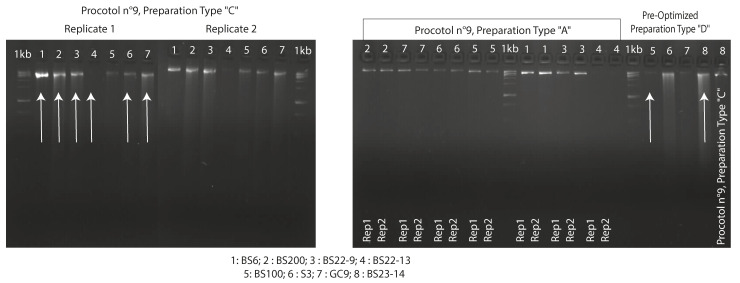
Agarose gel (2%, *w*/*v*) of gDNA obtained from eight strains. White arrows indicate the eight selected gDNA samples used for whole-genome sequencing with NextSeq550 Illumina platform.

Another major issue was identifying whether there is a difference between a fresh preparation (preparation type A); fresh, rinsed, squeezed and frozen preparation (C); or cryogenic mycelia treatment (preparation type D) using kit 5’s pre-optimized protocol. The control measurements of all gDNA extracts were obtained at different times (see Table S6 and Table 1, and Figure 5 and Figure 6).

To prove the efficiency, the reproducibility and the repeatability of our optimized protocol, we made first four replications for two different strains (BS6 and BS200) with kit 5’s manufacturer’s instruction (see Table S6c) and then two replications for each studied strain to make a final decision between fresh (preparation A) or frozen biomass (preparation C). Due to resource limitations, we only applied one replica per strain to finalize the optimization (see Table S6d). The eight fungal strains were treated as previously described and their genomic DNA extracted using the pre-optimized protocol to distinguish the tested preparations and further optimize the protocol. With over 0.3 g of mycelia yield treated with preparation D’s steps, the gDNA was of better quality and produced a higher quantity using the optimized protocol (available at protocols.io: https://doi.org/10.17504/protocols.io.8epv5r6yjg1b/v1) (see Table S6e).

### 3.2. PCA Amplification and Taxonomic Annotation

**Figure 7:**
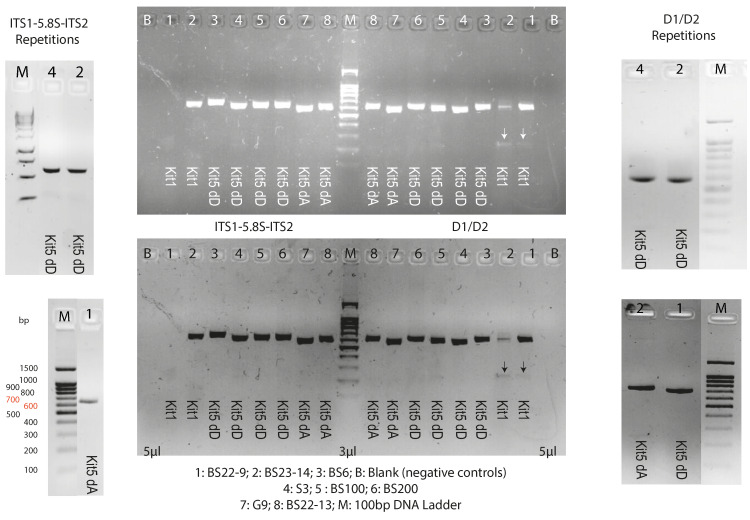
Agarose gel (1.5% *w*/*v*) of ITS and D1/D2 regions amplified for eight strains depending on the used extraction method. White arrows (on the negative image) and black arrows (on the positive image) indicate the contamination or non-specific products obtained with Phenol-Chloroform extracts compared to clean, single-band PCR products demonstrate high specificity and absence of cross-contamination or off-target amplification using gDNA samples extracted with our pre-optimized protocol.

To evaluate the suitability of the gDNA extracts, the selected samples were used for PCR analysis. The negative controls confirmed the specificity of the PCR program for both ITS region and D1/D2 domain amplifications. The results showed clearly that, for all the strains and amplified domains, the optimized protocol (D method) of the DNeasy Plant Pro Kit (kit 5) applied to samples with cryogenic treatment (sample preparation D) is the most suitable for amplification (Figure 7).

A sequence homology search using BLAST showed high identity levels with the core_nt database, ranging from 91.42% to 99.28% for ITS1-5.8S-ITS2 and from 96.98% to 95.89% for the D1/D2 regions (see Table S7 and Figure S3).

**Figure 8:**
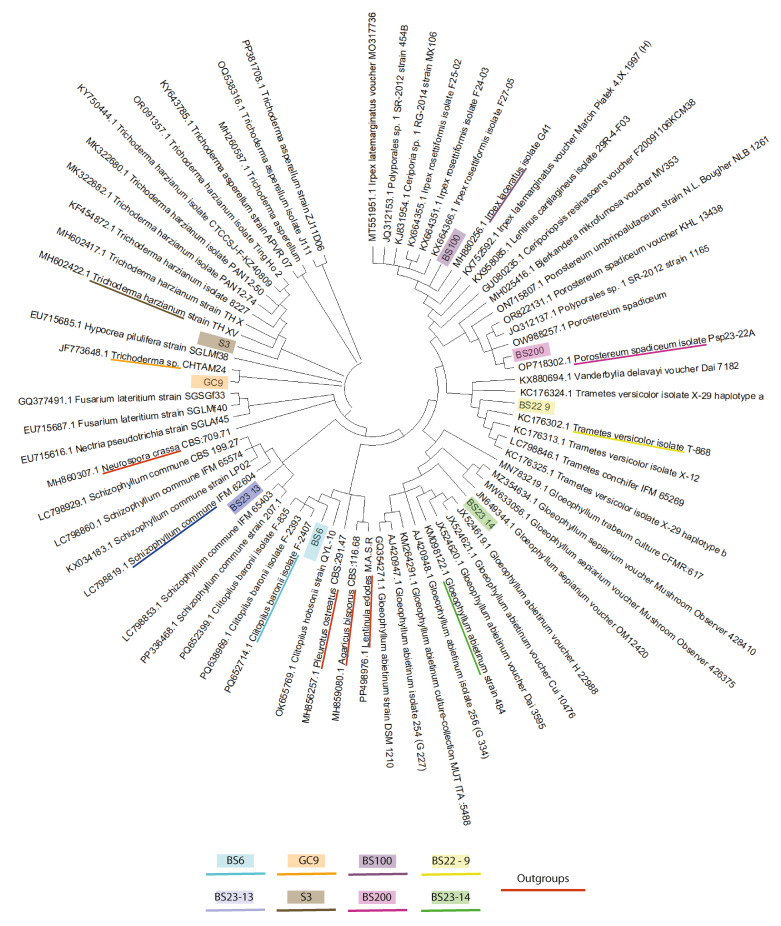
The maximum likelihood tree of the novel species and related taxa based on the rDNA ITS region and 28S rDNA D1/D2 domain combined sequences.

The combined sequences between the ITS1-5.8S-ITS2 region (561–606 bp) and D1/D2 region (554–601 bp) (a total between 1118 bp and 1207 bp) of the eight macrofungal strains were compared to the listed nucleotide sequences in Table S2. The phylogenetic trees provided the same taxonomic annotation to that obtained using the BLAST search, except for GC9 (Figure 8).

Based on these results, in addition to their morphological characteristics, the newly isolated strains were assigned to the *Clitopilus baronii* (BS6), *Porostereum spadiceum* (BS200), *Trametes versicolor* (BS22-9), *Schizophyllum commune* (BS23-13), *Gloeophyllum abietinum* (BS23-14), *Irpex laceratus* (BS100), *Trichoderma asperellum* (GC9) and *Trichoderma harzianum* (S3) species.

### 3.3. gDNA Concentration, Purity, Yield and Short- or Long-Read Sequencing Applications

To minimize subjective evaluation bias, we chose to compare the different protocols by referring to basidiomycetes. DNA yields varied depending on the method: with approximately 0.47 ± 0.05 g and 0.23 ± 0.1 g of filtered wet mycelium, QIAamp DNA Microbiome and Qiagen DNeasy Plant Pro produced 92 ± 11.31 ng/µL and 54.5 ± 7.78 ng/µL of genomic DNA, respectively, with 2.22 ± 0.04 and 1.92 ± 0.04, and 1.93 ± 0.75 and 1.75 ± 0.07 for 260/280 and 260/230, while approximately 1 g of biomass was required to obtain 90.5 ± 10.04 ng/µL of DNA with 1.97 ± 0.13 and 1.36 ± 0.67 for ratios of 260/280 and 260/230, respectively, using the phenol–chloroform method. Plant/fungal DNA isolation and plant genomic DNA purification kits exhibited the lowest yields. Across the tested *Basidiomycota* mycelium strains, using the DNeasy Plant Pro Kit with the manufacturer’s instructions without adding PS solution yielded the highest quantity of gDNA with acceptable purity ratios using a minimum amount of biomass compared to in-house method or other kits. The Qiagen DNeasy Plant Pro is therefore the most suitable for our fungus in our experimental context, which allows us to establish an optimized final protocol, as previously described (see Figure S2 and Table S6).

In fact, gDNA extraction from BS23-14 with the phenol–chloroform method provided a high yield with a Nanodrop concentration of 210.7 ng/μL and absorbance purity ratios of (2.12; 2.14), but the concentration estimated by Qubit was low (36.4 ng/μL). However, when extraction was done using kit 5 with the manufacturer’s instructions (see Table S1, Protocol n°7), the gDNA concentration measurements were similar between Nanodrop and Qubit. The lowest gDNA yield was for BS22-13 (0.31 ng/μL), and BS6 presented the highest value (11.83 ng/μL) for the same applied protocol (see Table S1, Protocol n°9) and sample preparation type C. The gDNA was pure for all strains treated with DNeasy Plant Pro Kit (kit 5), with values equal to 1.8 for a 260/280 ratio. However, phenol–chloroform yielded a higher pure gDNA value (A260/230 = 2.14), in contrast to extracts with kit 5 that showed lower values, especially for BS22-13, GC9 and S3 (see Table S8).

To ensure the stability of the gDNA control results before sequencing the samples, the selected samples were remeasured using Nanodrop and Qubit. There was an interesting decrease in values related to the two extractions based on preparation B. Even though the phenol–chloroform in-house protocol is a well-known gDNA extraction method (1820 μg DNA yield), our optimized protocol needed less input yield, 1/10, to produce approximately double the gDNA yield (2720 μg). Between preparations C and D, and with biomass yield ranges between 0.106 and 0.228 g, the gDNA yield values were quite variable using the pre-optimized protocol (see Table S8).

Nevertheless, regardless of the conditions and the protocol applied, the strains *Porostereum spadiceum* (BS200), *Clitopilus baronii* (BS6) and *Trametes versicolor* (BS22-9), which belong to the *Basidiomycota* phylum, often exhibited the highest gDNA yield values. But also, the pre-optimized protocol showed its potential power to extract an amount of gDNA yield (2720 μg) much closer to the maximum value than that obtained by kit 1 (1820 μg) for BS23-14 (see Table S8).

As Illumina technologies can manage more gDNA extracts with the phenol–chloroform method than ONT; BS23-14 (kit 1, B-type) was sequenced with the MiSeq platform (see Table S8 and Figure 5A at the bottom). On the other hand, Mk1C was the sequencing platform chosen for BS6 gDNA extracted with kit 5 using the manufacturer’s instructions and preparation type B (see Table S8, Figure 5B). The same strain was again sequenced with the same technology, but its gDNA was treated with the pre-optimized protocol for kit 5 and sample preparation type C (see Table S8, Figure 5C).

Finally, all eight strains treated with the pre-optimized protocol for kit 5 and with sample preparation type C, except for BS100 and BS23-14, whose mycelia were cryogenically treated (D), were chosen to be sequenced with high-throughput technologies (see Table S8 and Figure 6 highlighted with white arrows).

### 3.4. Raw Data Bioinformatics Analyses

The size of the reads after the quality check were 2.4 Gb, 36.7 Gb, 806.5 Mb and 148.94 Mb for MiSeq (>Q30), Nextseq550 (>Q30) and Mk1C (>Q9), respectively. The depth of the reads ranged from 3.7× to 83× depending on the used sequencing platform (see Table S9).

For a comprehensive comparison of methods and final recommendations regarding the protocol, we performed standard bioinformatics analysis on the raw data collected from different fungal strains to compare assembly metrics (N50, contig count) and genomic quality across extraction methods, while considering relevant differences between sequencing technologies (see Tables S4 and S10–S13).

The assembled genomes’ total lengths (with ≥1000 bp cutoff) ranged from 29,209,248 bp to 53,385,455 bp. Their scaffold length was between 454.22 and 22,278.43 bp, 453.40 bp, and 16,097.01 and 47,036.60 bp for Nextseq550, Miseq and Mk1C, respectively. The largest scaffold of all the analyzed genomes was obtained for the S3 strain (2,577,683 bp) using Nextseq550 followed by BS6 with the first run of Mk1C (1,332,670 bp). All the analyzed strains’ genome assemblies had a relatively high GC content, ranging from 47.37% (GC9) to 57.48% (BS22-9) (see Tables S10 and S11).

QUAST metrics demonstrated that N50 and L50 ranged from 911,563 bp as the maximum to 2,575 bp as the minimum and from 15 to 3,476 for *Trichoderma harzianum* S3 and BS22-9, respectively. Despite the technical issue with the second MinION run, its smallest scaffold (541 bp) was longer than that observed in the first run (246 bp) using gfastats metrics, which systematically approve the quality of the sequenced sample obtained from the pre-optimized protocol and sample preparation C. Comparing the gfastats/QUAST metrics of BS23-14 between the two different Illumina platforms used, the assembled genome of gDNA extracted with phenol–chloroform/B (MiSeq) gave values of 138,133 and 78 bp, less significant than 347,930 and 56 bp obtained from the pre-optimized protocol/D with NextSeq550. Thus, the pre-optimized protocol with cryogenic treatment is more suitable for selection than any other protocol or sample preparation, especially by looking at the obtained total gap length in scaffolds (bp), which is equal to 6420 and 218,086, respectively, of each assembled genome. After pre-validating the pre-optimized protocol using the QIAGEN DNeasy Plat Pro Kit, it is now crucial to decide between using simply frozen, well-conserved mycelium (C) or cryogenically treated mycelium (D). Taking into consideration the differences between basidiomycetes and ascomycetes, BS100 and BS23-14, which were treated with D, gave the two largest scaffolds (795,057 and 347,930 bp, respectively) compared to the other basidiomycetes strains (BS6, BS200, BS22-9, and BS22-13) treated with C (66,855–196,398 bp) (see Tables S10 and S11).

The BandageInfo metrics summarized in Table S12 demonstrate high node counts (779–121,225) and dead ends (272–7366) (0.16–94.22%), proving the fragmented aspect of our different assembled genomes using a default and common bioinformatics pipeline without any further optimization either of hyperparameters or even of the used tools. Overall, the BandageInfo results present many potential paths through the different assemblies regardless of the sequencing technique used or the applied protocol for gDNA extraction. However, the longest obtained node was equal to 1,327,226 bp with the first Mk1C run for the BS6 strain (see Table S12).

Among the sequenced strains with Nextseq550, the longest N50 scaffold was for the S3 strain with 911 Kb, followed by GC9 (223 Kb) and BS23-14 (37 Kb), and it ranged between 2 Kb and 37 Kb for all other strains. This observation highlights the impact of the pre-optimized protocol with sample preparation D on downstream analyses, except for S3, whose biomass was used as described in treatment C. Both N50 of the *Basidiomycota* strains were longer than the other species (2–7 Kb) treated with sample preparation C. The N50 scaffold of the BS6 strain was equal to 6 Kb with Nextseq550 and 204 Kb with the first run of Mk1C. However, Nextseq550 gave a greater N50 (37 Kb) than MiSeq (6 Kb) for the BS23-14 strain, which is related to the extraction method (optimized protocol), a rarely quantified link. The BUSCO analysis confirmed that S3 and GC9 are *Ascomycota* and the other strains are *Basidiomycota* based on complete and single-copy (S) identification compared to the adequate databases, which affirms more strongly the phylum of the different strains as described previously. Despite a 688 Kb difference between their best N50 scaffolds for NextSeq550, S3 and GC9 have almost the same number of single-copy genes (S), with duplication (D) equal to five and three, respectively (see Table S13).

## 4. Discussion

A total of eight different fungal Tunisian species, selected from a private ancient collection, were included in our study, with two and six species from the *Ascomycota* and *Basidiomycota* phyla, respectively. Among the five methods used and the four different sample preparations, the optimized DNeasy Plant Pro (kit 5) protocol using the cryogenic sample preparation (D) was found to be the best approach. If the biological material is ready for use (culture, filtration, and cryogenic treatment), our optimized protocol allows us to obtain gDNA in less than an hour with a quantity and quality suitable for both PCR amplification and high-throughput sequencing (short and/or long reads).

Our findings are consistent with Huang et al. (2018) reporting that a liquid nitrogen grinder is the best applied technique to destroy the cell wall [30]. As a well-known mechanical technique for fungal gDNA extraction, cryogenic treatment allows us to improve the quality and yield of the samples [31]. However, Riffiani et al. (2021) [32] used the DNeasy Plant Pro (or Mini) Kit as a second step to purify their extracted gDNA with the classic Cetyltrimethylammonium Bromide (CTAB) method from mycelium culture. Although Riffiani et al. (2021) refined the culture medium to achieve the best composition and maximize gDNA extraction, we obtained similar quantities with minimal culture medium and without any optimization of culture conditions [32].

However, it is known that fungal DNA can be highly affected during collection, transport or during extraction due to shearing [33]. The DNA migration profile of the different extractions is quite varied and depends on the protocol used. The amount of collected gDNA using the in-house protocol (kit 1) was different between the two used strains (BS23-14 and BS22-9). For both BS6 and BS200, the pre-optimized protocol (n°9) using sample preparation D was more accurate than using fresh culture. But overall, our findings show that preparation D is the most likely to have a better band and a good quality and quantity of gDNA even with the shearing aspect.

When considering the criteria for choosing the best protocol, one must not forget the difference observed between the DNA concentration values depending on the tool used. The observations demonstrate that the Nanodrop measurement is higher than Qubit’s in the case of the gDNA extracted with phenol–chloroform for BS23-14, which was not the case when using kit 5 with the manufacturer’s instructions. Indeed, residual phenols, proteins, and even compounds released from plastic tubes during storage can interfere at 230–260 nm without any proportional DNA/RNA existence. Therefore, various studies have demonstrated the difference between UV-based quantification and fluorescence-based assays, especially in the presence of extraction reagents and/or UV-absorbing contaminants. The large discrepancy between Nanodrop and Qubit is due to the UV absorbance bias rather than true nucleic acid content, as fluorometric methods rely on dyes reflecting the amount of material available for downstream analysis. Qubit thus reports lower but functionally more relevant double-stranded nucleic acid. It is then more selective and insensitive to phenol and co-extracted impurities than Nanodrop. Consequently, protocol optimization and downstream normalization should preferentially be based on fluorometric measurements [34,35].

It is commonly know that the in-house phenol–chloroform protocol is effective in removing proteins, hydrophobic peptides, and cell debris due to phenol use. But because of its toxicity and interference with yield estimation and downstream enzymatic processes, it is crucial to eliminate it from gDNA samples [33]. However, recent fungal gDNA extraction methods rely on the Qiagen DNeasy Plant Mini/Pro Kit [36]. Feng et al. (2010) [36] compared the Invitrogen PureLink Genomic DNA Mini Kit, Qiagen DNeasy Plant Mini Kit and a personalized protocol to extract high-quality DNA from 26 fungal species (0.02 g of biomass) for PCR amplification. They found that the highest amount of gDNA was obtained from *Botrytis cinerea*, an ascomycete strain, compared to their specific protocol [36].

Trying to evidence the reproducibility of our optimized protocol, the validation step was done in a different environmental condition and at another time period. Using mycelia yields spanning between 0.391 g and 0.485 g for PCR or/and high-throughput sequencing purposes, our optimized protocol gave gDNA concentrations between 10.1 and 444 ng/μL (Qubit values) and an A260/280 ratio of 1.78–1.97, which perfectly match with Feng et al.’s (2010) work in terms of A260/280 ratios found using the Qiagen DNeasy Plant Mini Kit [36]. Finally, our study proves that the collected gDNA increases by increasing the amount of biomass used as input for our optimized protocol, independently of the studied strain.

Thus, our optimized protocol, using the commercial kit DNeasy Plant Pro on a cryogenically pretreated fungal mycelium, is user-friendly and constitutes a faster, safer, more cost-efficient and less toxic protocol than the phenol–chloroform method, while guaranteeing results (protocols.io: https://doi.org/10.17504/protocols.io.8epv5r6yjg1b/v1; see Table S14).

PCR amplification was the first downstream check analysis for the extracted gDNA. Regardless of the strain and its unknown secondary metabolites, the ITS1-5.8S-ITS2 and D1/D2 regions were reliably amplified by PCR across all gDNA samples, proving the absence of inhibitor substances (DNases and other enzymes [37]) that could interfere with downstream experiments or analyses. When each PCA product was electrophoresed to confirm quality, a sharp band was produced for each amplified sample from gDNA extracted with our optimized protocol, especially compared to samples extracted with the phenol–chloroform protocol (kit 1), indicating that extractions from our optimized protocol produced gDNA of suitable quality for PCR amplifications.

While NanoDrop confirms purity, functional testing (e.g., serial dilution PCR of 1:10 and 1:100) verifies inhibitor removal. We further recommend that users perform this for critical applications if samples are obtained. Therefore, the DNeasy Plant Kit’s efficacy against fungal polysaccharides, polyphenolics, and other secondary metabolites from plant/fungal biomass is ensured by QIAGEN’s patented Inhibitor Removal Technology [38].

The phylogenetic analyses were conducted using four different known mushroom species (*Neurospora crassa* (*Ascomycota*), *Lentinula edodes* (*Basidiomycota*), *Pleurotus ostreatus* (*Basidiomycota*), and *Agaricus bisporus* (*Basidiomycota*)) as an outgroup of genus and species. *Clitopilus baronii* (BS6) was the closest strain to them, except for *Neurospora crassa*, which is obvious since our strain belongs to the phylum of basidiomycetes, while the reference is ascomycetes. BS23-14 was identified as *Gloeophyllum abietinum*, but it was kind of distinguishable from the compared strains despite the existence of other species from the same genus. This suggests that our strain is probably different. The same hypothesis applies for *Irpex laceratus* (BS100), *Trametes versicolor* (BS22-9), *Trichoderma harzianum* (S3) and *Schizophyllum commune* (BS23-13). The closest strain to BS200 identified on the phylogenetic tree was *Porostereum spadiceum* isolate Psp23-22A (OP718302.1). Nevertheless, the identification of GC9 was mainly based on separate ITS and D1/D2 phylogenetic trees as *Trichoderma asperellum*.

*Trametes versicolor* [39], *Trichoderma harzianum* [40], *Schizophyllum commune* [41], *Trichoderma asperellum* [42,43] and *Porostereum spadiceum* [44] are known and sequenced species. However, *Clitopilus baronii*, *Gloeophyllum abietinum* and *Irpex laceratus* are probably novel unsequenced genomes.

This study has numerous limitations that should be highlighted. First, we evaluated only eight privately owned fungal strains, which does not allow us to fully capture the taxonomic and morphological diversity of fungi. Second, the replication of extraction protocols was inconsistent due to practical constraints related to the availability of samples and reagents, which likely affects the robustness of exhaustive pairwise comparisons. Third, these methodological limitations restrict our ability to perform a formal statistical analysis for all the protocols applied; therefore, the observed differences should be interpreted as indicative trends rather than definitive ranking.

While boxplot visualizations offer descriptive insights into yield/purity distributions, they are not a substitute for inferential statistics. Comparative evaluations of fungal gDNA extraction methods remain common in the recent high-impact literature. For example, Langsiri et al., 2025, compared spin-column and bead-based methods for clinical fungal isolates using yield and purity metrics without formal statistical analysis, focusing instead on practical sequencing like our comparison approach [31].

Nevertheless, aware of these limitations, we sought to mitigate them by performing PCR amplification of ITS and D1/D2 regions from the gDNA obtained (as described previously) and high-throughput sequencing to compare the performance of the following finally selected protocols: phenol–chloroform, Qiagen DNeasy Plant Pro (with the manufacturer’s instructions), the pre-optimized Qiagen DNeasy Plant Pro protocol and optimized final protocol. This shortcoming notwithstanding, functional assays (PCR and NGS) substantiate the selected protocol’s efficacy.

In fact, the selected gDNA samples from each strain, obtained mainly from two unlike protocols (kit 1 or kit 5) and three different biomass preparations (B, C and D), were chosen to deeply evaluate the efficiency of the most appropriate protocol (1, 7 or 9) through the calculated metrics using bioinformatics analyses, after being sequenced using Illumina and/or ONT, whether for resequencing known genomes or de novo genomes.

Using MiSeq, Nextseq550, MinION Mk1C—1st, and MinION Mk1C—2nd, we achieved 60×, 83×, 20× and 3.7× per sample for phenol–chloroform/fresh and filtered mycelium (sample preparation B); QIAGEN DNeasy Plant Pro Kit/sample preparation B with the manufacturer’s instructions; pre-optimized protocol/fresh, rinsed, squeezed, and frozen mycelium (sample preparation C); and pre-optimized protocol/sample preparation C or cryogenic treatment (sample preparation D), respectively, as a raw data depth values.

Overall, obtaining 20× for long reads with the pre-optimized protocol/sample preparation C compared to 3.7× in the second run is crucially due to a technical problem related to flow cell failure just after 16 h (starting nanopore number less than 950) compared to the first run, with approximately 1300 nanopores which were ruined at 72 h. However, getting 60× relies on running one single sample on Miseq compared to eight samples (plus three others not mentioned in this work) on NextSeq550, which gave a theoretical coverage of around 83× for each sample. Mittelstrass et al., 2025, demonstrated that long-read sequencing technology could enhance species and genus identification but would probably reduce sequencing depth, thus limiting diversity coverage and giving a higher error rate compared to Illumina platforms [45]. The dramatic variation between the depth of coverage values of the Illumina platforms and the Mk1C device is therefore due to the platform throughput characteristics and the kits used rather than sample quality.

The highest level of GC content belongs to *Trametes versicolor* BS22-9 (57.48%) but *Trichoderma asperellum* GC9 has the lowest percentage with 47.37%, values that perfectly match with the literature values equal to 57.68% and 47.38%, respectively [39,43]. The total length (≥1000 bp) ranges between 29.2 Mbp and 53.4 Mbp, where the highest belongs to BS22-9, slightly different to Yang and Hu’s (2025) [39] results (47.42 Mbp), and the lowest to *Irpex laceratus* BS100. The latter’s length totally disagrees with recent studies, which refer to its closest genome *Irpex lacteus*, which reported an average of 44.4 Mbp [46,47], thus proving the novelty of our strains.

*Basidiomycota* and *Ascomycota* are well-known fungal phyla, with their highly diverse secondary metabolism (polypeptides, acetylenes, etc.) having antioxidant, antimicrobial, anti-inflammatory and other potential effects and their cellulolytic activity system to degrade lignocellulosic materials [43,48,49].

However, these findings reflect laboratory conditions with a limited strain panel and uneven replication, and the optimized protocol’s effectiveness may vary across fungal taxa with different cell wall compositions or growth conditions. While not standardizing to all fungi, the observed advantages of the optimized protocol—confirmed through functional PCR and sequencing read and genome assembly quality—offer practical value for laboratories working with similar species.

The Tunisian strain collection represents underrepresented fungal biodiversity. Phylogenetic analyses reveal extraction challenges correlated with cell wall composition, as documented in regional phylogenetic surveys [50]. Cryogrinding-optimized DNeasy Plant Pro presented particularly effectiveness for our eight distinct and phylogenetically distant strains. Our study offers global genomics researchers a framework to anticipate extraction needs based on fungal morphology and evolutionary relationships. Given the importance of a thorough characterization of the genome and the genes potentially present in our eight phylogenetically distinct strains, further bioinformatic analyses and optimization are strongly recommended.

## 5. Conclusions

This study was motivated by the availability of an unexplored private collection, from which eight different macrofungal strains belonging to two phyla were sampled. Given the diverse industrial applications of this selection, our primary goal was to evaluate the reproducibility, efficiency, and time/cost optimization of genomic DNA extraction protocols through several control analyses. To achieve this, we refined a protocol based on the Qiagen DNeasy Plant Pro Kit (kit 5) and cryogenically treated the biomass as a pretreatment (sample preparation D) to ensure a good yield. Our optimized method is adequate, easy to implement, and user-friendly involving basidiomycetes and ascomycetes. Our findings prove that the extracted gDNA can be used in downstream applications such as PCR amplification or even second- or third generation sequencing. We do not claim universal applicability to all sample types, as extraction efficacy remains dependent on the matrix (mycelium, fruiting body, environmental sample, etc.). Our comparative evaluation therefore provides practical guidance for selecting and optimizing the most appropriate fungal gDNA extraction method in similar laboratory settings, while acknowledging that the limited number of strains, uneven replication, and lack of comprehensive statistical analysis restrict the scope of the experimental design. Future work including a larger and more diverse panel of strains, balanced replication, and a fully factorial design will be important to confirm and extend these findings.

## Figures and Tables

**Table 1:** List of the selected pure fungal Tunisian strains.

Strains	Phyla	Genus	Species	Origins	Collection Dates	Figure Legends	
						1	2a
BS6	*Basidiomycota*	*Clitopilus*	*baronii*	Pieces of decaying wood	November 2021	-	D
BS200	*Basidiomycota*	*Porostereum*	*spadiceum*		November 2021	D	G
BS22-9	*Basidiomycota*	*Trametes*	*versicolor*		March 2022	-	F
BS23-14	*Basidiomycota*	*Gloeophyllum*	*abietinum*		March 2023	A & B	H
BS22-13	*Basidiomycota*	*Schizophyllum*	*commune*		March 2022	C	E
S3	*Ascomycota*	*Trichoderma*	*harzianum*		Winter 2010	-	A
BS100	*Basidiomycota*	*Irpex*	*laceratus*			-	B
GC9	*Ascomycota*	*Trichoderma*	*asperellum*	Chemical group effluent	May 2022	-	C

**Table 2:** General experimental design.

Protocols *	Selected gDNA Extraction Methods			Criteria			Sample Preparations			
				S	C	T ^c^	A	B	C	D
1	Phenol–Chloroform (kit 1) ^a^			*	***	*		√		
2	QIAamp DNA Microbiome (kit 2) ^b^	a	Manufacturer’s instructions	***	*	***		√		
3		b	Alumina	***	*	***		√		
4		c	Optimized	***	*	***		√		
5	Plant/Fungi DNA Isolation (kit 3) ^b^		Manufacturer’s instructions	***	***	***		√		
6	Genomic DNA Purification Plant (kit 4) ^b^		Manufacturer’s instructions	**	**	***		√		
7	DNeasy Plant Pro (kit 5) ^b^	a	Manufacturer’s instructions	***	**	***	√	√		
8		b	With PS Solution	**	**	***		√		
9		c	Pre-Optimized	***	**	***			√	√
10		d	Optimized **	***	**	***				√

* The number of the protocol in the Supplementary Materials (see Table S1); ** the final optimized protocol is meticulously detailed at protocols.io (https://doi.org/10.17504/protocols.io.8epv5r6yjg1b/v1) and visually represented in Figure 3. ^a^ In-house protocol by Aifa et al. (1999) [19]; ^b^ commercial; ^c^ needed time to process 10 samples. **Safety (S)**: * low, ** medium, and *** high. **Cost (C)**: * high, ** medium (acceptable for most labs), and *** low. **Time (T)**: * long (>120 min), ** moderate ([90–120]), and *** short (≤90). **A**: Sterile fresh mycelia, directly extracted (Figure S1a); **B**: fresh and filtered mycelia, stored at 20 °C (Figure S1c); **C**: fresh, rinsed, squeezed, and frozen mycelia at −20 °C (Figure S1b—A and B); **D**: treated as “C” and turned into fine powder with liquid nitrogen to be stored at −20 °C (Figure S1b—A–D).

## Data Availability

Newly generated sequences generated in this study are available in the National Center of Biotechnology Information GenBank databases, following the accession numbers in the manuscript. All other data are included in the article or Supplementary Materials. Further inquiries can be directed at the corresponding author. The DOI of the optimized selected protocol mentioned in the article paper is available at protocols.io: https://doi.org/10.17504/protocols.io.8epv5r6yjg1b/v1.

## Supplementary Materials

The following supporting information can be downloaded at: https://www.mdpi.com/article/10.3390/jof12050299/s1, Table S1: List of applied protocols and related steps; Figure S1: Sample preparation methods; Table S2: Used GenBank accession number sequences in molecular phylogenetic analysis; Table S3: Applied NGS technologies during this study on the validated gDNA fungal samples; Table S4: Tools used in the bioinformatics analyses; Table S5: List of nucleotide sequence and whole genome sequence accession numbers of the eight Tunisian fungal strains; Table S6: gDNA concentration and purity obtained from different applied protocols; Figure S2: Comparison between different applied protocols in terms of biomass, purity (260/280 & 260/230) and yield (qubit) in the first phase (see Table S6a); Table S7: BLAST algorithm detailed report Molecular identification of newly isolated medical mushrooms by ITS1-5.8S-ITS2 and D/D2 region of rRNA gene sequence analysis; Figure S3: ITS and D1D2 phylogenetic trees; Table S8: gDNA concentrations, purity ratios and yield in each chosen sample for sequencing; Table S9: Raw data metrics obtained from the different sequencing technologies used (Miseq, Nextseq550 & Mk1C); Table S10: Summarize of the assembled genomes’ quality control sequenced with Mk1C and Miseq; Table S11: Summarize of the eight assembled genomes’ quality control based on gfstats/QUAST metrics for NextSeq550 outputs; Table S12: BandageInfo results of the different assembled genomes; Table S13: BUSCO results of the different assembled genomes; Table S14: Cost and risk analysis between phenol–chloroform and our optimized protocol.

## Abbreviations

The following abbreviations are used in this manuscript:

rDNA	Ribosomal DNA
DNA	Deoxyribonucleic Acid
gDNA	Genomic DNA
RNA	Ribonucleic Acid
NGS	Next-Generation Sequencing
ITS	Internal Transcribed Spacer
PDA	Potato Dextrose Agar
LSU	Large SubUnit
PCR	Polymerase Chain Reaction
UV	Ultra-Violet
BLASTN	Basic Local Alignment Search Tool for Nucleotide
MEGA	Molecular Evolutionary Genetics Analysis
ONT	Nanopore Oxford Technologies
WGS	Whole Genome Sequencing
CTAB	Cetyltrimethylammonium Bromide
