# Supplementary material for: Impact of DNA Extraction Strategies on Genomic and Bioinformatic Outcomes in Eight Selected Fungal Strains
Source: J Fungi (Basel). 2026 Apr 22;12(5):299. doi: 10.3390/jof12050299 (PMC13208813; doi:10.3390/jof12050299)
Supplement: Supplementary file 1 [file jof-12-00299-s001.zip › jof-4160139-supplementary.pdf]

Supplemental Table S1. List of applied protocols and related steps

Protocol n° 1. *Kit n°1* – Classical Phenol/Chloroform Extraction $_{g}$DNA Protocol

1. Weigh 2g of frozen mycelium.
2. Add 2g of alumina.
3. Grind to a powder in a mortar.
4. Add 5ml lysis buffer.
5. Recover the volume in a falcon tube.
6. Add a further 5ml of lysis buffer to recover the remainder.
7. Add 10ml Phenol/Chloroform.
8. Centrifuge 5000rpm/7min.
9. Recover upper phase.
10. Add 10ml Phenol/Chloroform.
11. Centrifuge 5000rpm/7min.
12. Recover supernatant and add 1V isopropanol.
13. Incubate 20 minutes at room temperature.
14. Centrifuge for 10 minutes at 6000rpm.
15. Wash with 70% ethanol.
16. Recover pellet in approx. 120µl of sterile distilled water.

Protocol n° 2. *Kit n°2.a* - QIAamp DNA Microbiome Kit

1. Add 500µl Buffer AHL to 1ml of sample in a 2ml tube (not provided) and incubate for 30min at room temperature with end-over-end rotation.
2. Centrifuge the tube at 10 000g for 10min and remove the supernatant.
3. Add 190µl of Buffer RDD and 2.5µl of Benzonase. Mix well and incubate at 37°C for 30min at 600rpm in a heating block or water bath.
4. Add 2µl Proteinase K and incubate at 56°C for 30min at 600rpm in a heating block or water bath. Then, briefly spin the tube at slow speed.
5. Add 200µl Buffer ATL (containing Reagent DX). Mix well and transfer into Pathogen Lysis Tube L. Place Pathogen Lysis Tube in the genomic vortex for 10 minutes with maximum speed.
6. Centrifuge Pathogen Lysis Tube L at 10 000g for 1min and transfer the supernatant into a fresh microcentrifuge tube. Add 40µl Proteinase K, vortex to mix and incubate at 56°C for 30 minutes in a heating block (vortex in between).
7. Add 200µl Buffer APL2. Mix by pulse-vortexing for 30s. Incubate at 70°C for 10min and briefly spin the tube.
8. Add 200µl ethanol to the lysate and mix by pulse-vortexing for 15-30s. Apply up to 700µl of the mixture to the QIAamp UCP Mini spin column without wetting the rim. Close the cap and centrifuge at 6 000g for 1min. Discard the flow-through and put the column back into the same collection tube. Repeat with any remaining ethanol-lysate mixture.
9. Transfer the QIAamp UCP Mini spin column to a fresh collection tube. Open the cap and add 500µl Buffer AW1 without wetting the rim. Close the cap and centrifuge at 6 000g for 1 min. Place the QIAamp UCP Mini spin column into a fresh 2ml collection tube and discard the filtrate.
10. Open the QIAamp UCP Mini spin column and add 500µl Buffer AW2^{+} without wetting the rim. Centrifuge at 12 000g for 6min.

**11.** Place the QIAamp UCP Mini spin column into a fresh 2ml collection tube and discard the filtrate. Centrifuge at 20 000g for 2min.<br>**12.** Place the QIAamp UCP Mini spin column into a fresh 1.5ml tube and apply 50µl Buffer AVE^{+} directly onto the center of the membrane. Close the lid and incubate at room temperature for 5min. Centrifuge at 6 000g for 1min to elute the DNA.
**Protocol n° 3.** *Kit n°2.b* - QIAamp DNA Microbiome Kit using alumina<br><br>**1.** Weigh 1g of frozen biomass (-80°C) and an equal weight of alumina (V/V ratio).<br>**2.** Grind in a pre-cooled mortar then add 5ml of Proteinase K buffer until a paste is obtained.<br>**3.** The collected volume is divided into Eppendorf tubes for possible centrifugation (maximum of 3 minutes). Treat the aliquots with proteinase K (1/10) and incubate at 65°C for 1 hour to 2 hours (dray bath, vortex in between).<br>**4.** Centrifuge at 12 000rpm and transfer the supernatant into a fresh microcentrifuge tube.<br>**5.** Add 200µl Buffer ATL (containing Reagent DX). Mix well and transfer into Pathogen Lysis Tube L. Place Pathogen Lysis Tube in the genomic vortex for 10 minutes with maximum speed.<br>**6.** Centrifuge Pathogen Lysis Tube L at 10 000g for 1min and transfer the supernatant into a fresh microcentrifuge tube. Add 40µl Proteinase K, vortex to mix and incubate at 56°C for 30 minutes in a heating block (vortex in between).<br>**7.** Add 200µl Buffer APL2. Mix by pulse-vortexing for 30s. Incubate at 70°C for 10min and briefly spin the tube.<br>**8.** Add 200µl ethanol to the lysate and mix by pulse-vortexing for 15-30s. Apply up to 700µl of the mixture to the QIAamp UCP Mini spin column without wetting the rim. Close the cap and centrifuge at 6 000g for 1min. Discard the flow-through and put the column back into the same collection tube. Repeat with any remaining ethanol-lysate mixture.<br>**9.** Transfer the QIAamp UCP Mini spin column to a fresh collection tube. Open the cap and add 500µl Buffer AW1 without wetting the rim. Close the cap and centrifuge at 6 000g for 1 min. Place the QIAamp UCP Mini spin column into a fresh 2 ml collection tube and discard the filtrate.<br>**10.** Open the QIAamp UCP Mini spin column and add 500µl Buffer AW2^{+} without wetting the rim. Centrifuge at 12 000g for 6min.<br>**11.** Place the QIAamp UCP Mini spin column into a fresh 2ml collection tube and discard the filtrate. Centrifuge at 20 000g for 2min.<br>**12.** Place the QIAamp UCP Mini spin column into a fresh 1.5ml tube and apply 50µl Buffer AVE^{+} directly onto the center of the membrane. Close the lid and incubate at room temperature for 5min. Centrifuge at 6 000g for 1min to elute the DNA.
**Protocol n° 4.** *Kit n°2.c* – Optimized steps using QIAamp DNA Microbiome Kit<br><br>**1.** Weigh 0.5g of frozen biomass into 2ml tube (not provided).<br>**2.** Thaw biological material at room temperature.<br>**3.** After thawing, draw the liquid into the tube and treat the sample with proteinase K (1/10) by adding 1ml of proteinase K buffer, and incubate at 65°C for 1 hour to 2 hours (dray bath, vortex in between).<br>**4.** Centrifuge at 12 000rpm and transfer the supernatant into a fresh microcentrifuge tube.

5. Add 200µl Buffer ATL (containing Reagent DX). Mix well and transfer into Pathogen Lysis Tube L. Place Pathogen Lysis Tube in the genomic vortex for 10 minutes with maximum speed.
6. Centrifuge Pathogen Lysis Tube L at 10 000g for 1min and transfer the supernatant into a fresh microcentrifuge tube. Add 40µl Proteinase K, vortex to mix and incubate at 56°C for 30 minutes in a heating block (vortex in between).
7. Add 200µl Buffer APL2. Mix by pulse-vortexing for 30s. Incubate at 70°C for 10min and briefly spin the tube.
8. Add 200µl ethanol to the lysate and mix by pulse-vortexing for 15-30 s. Apply up to 700µl of the mixture to the QIAamp UCP Mini spin column without wetting the rim. Close the cap and centrifuge at 6 000g for 1min. Discard the flow-through and put the column back into the same collection tube. Repeat with any remaining ethanol-lysate mixture.
9. Transfer the QIAamp UCP Mini spin column to a fresh collection tube. Open the cap and add 500µl Buffer AW1 without wetting the rim. Close the cap and centrifuge at 6 000g for 1 min. Place the QIAamp UCP Mini spin column into a fresh 2 ml collection tube and discard the filtrate.
10. Open the QIAamp UCP Mini spin column and add 500µl Buffer $AW2^{+}$ without wetting the rim. Centrifuge at 12 000g for 6min.
11. Place the QIAamp UCP Mini spin column into a fresh 2ml collection tube and discard the filtrate. Centrifuge at 20 000g for 2min.
12. Place the QIAamp UCP Mini spin column into a fresh 1.5ml tube and apply 50µl Buffer AVE^{+} directly onto the center of the membrane. Close the lid and incubate at room temperature for 5min. Centrifuge at 6 000g for 1min to elute the DNA.

Protocol n° 5. *Kit n°3* – Plant/Fungi DNA Isolation Kit - NORGEN Biotech Corp

Lysate preparation
1. Place ≤100 mg of plant tissue or wet fungi into a mortar that contains liquid Nitrogen and grind it into a powder. Transfer the plant or fungi powder to a DNase-free 1.7ml microcentrifuge tube (not provided) and add 500 µl of Lysis Buffer L and 1 µl of RNAse A.
2. Incubate at 65°C for 10 minutes. Occasionally mix the lysate 2- or 3-times during incubation by inverting the tube.
3. Add 100µl of Binding Buffer I, mix thoroughly and incubate for 5 minutes on ice.
4. Assemble a filter Column (clear O-ring) with one of the provided collection tubes. Pipette the lysate into Filter Column and spin for 2 minutes at 14 000g (14 000rpm).
5.Transfer only the clear supernatant from the flow-through into a DNase-free microcentrifuge tube (not provided) using a pipette.
6. Add an equal volume of 70% ethanol (provided by the user) to the lysate collected above (100µl of ethanol is added to every 100µl of lysate). Vortex to mix.

Binding to Column
1. Assemble a Spin Column (grey O-ring) with one of the provided collection tubes.
2. Apply up to 650µl of the clarified lysate with ethanol onto the Spin Column and centrifuge for 1 minute at 10 000g (10 000rpm). Discard the flow-through and reassemble the spin column with the collection tube.
3. Depending on your lysate volume, repeat step 2 if necessary.

Column Wash
1. Apply 500µl of Solution WN to the column and centrifuge for 1 minute.

2. Discard the flowthrough and reassemble the spin column with its collection tube.
3. Apply 500µl of wash Solution A to the column and centrifuge for 1 minute.
4. Discard the flowthrough and reassemble the spin column with its collection tube.
5. Repeat Step 3 and 4.
6. Spin the column for 2 minutes at 14 000g (14 000rpm) to thoroughly dry the resin. Discard the collection tube.

DNA Elution

1. Place the column into a fresh 1.7ml Elution tube provided with the kit.
2. Add 100µl of Elution Buffer B to the column and incubate for 1 minute at room temperature.
3. Centrifuge for 1 minute at 10 000g (10 000rpm). Note the volume eluted from the column. If the entire volume has not been eluted, spin the column at 14 000g (14 000rpm) for 1 additional minute.
4. (Optional): an additional elusion may be performed if desired by repeating steps 2 and 3 using 50µl of Elution Buffer in a different elution tube. The total yield can be improved by an additional 20-30% when this second elution is performed.

Storage DNA

The purified genomic DNA can be stored at 2-8°C for a few days. For longer term storage, -20°C is recommended.

Protocol n° 6. Kit n°4 – Genomic DNA Purification Plant - Molecular Biology Kit – BioBasic

1. Grind 100mg fresh plant tissue (or 20mg dry plant tissue) to fine powder in liquid nitrogen. Transfer the powder to a 1.5ml tube.
2. Add 600µl Buffer PCB and 12µl of β-mercaptoethanol to the sample and mix thoroughly by vertexing. Incubate at 65°C for 25min.
3. Add 0.6ml of chloroform to the tube, mix well by inverting 10 times. Centrifuge at 12 000 g for 2 minutes. Carefully transfer the supernatant (400µl) to a clean 1.5ml tube.
4. Add 200µl Buffer BD, mix thoroughly by vertexing.
5. Add 200µl ethanol (96-100%), mix thoroughly by vertexing.
6.Transfer the mixture from step 5 (including any precipitate) into the EZ-10 column placed in a 2ml collection tube. Centrifuge at 9 000g (12 000rpm) for 1min. Discard the flow-through.
7. Add 500µl PW Solution, and centrifuge for 1min at 9 000g (12 000rpm). Discard the flow-through.
8. Add 500µl Wash Solution, and centrifuge for 1min at 9 000g (12 000rpm). Discard the flow-through.
9. Place the empty column in the microcentrifuge and centrifuge for an additional 2min at 9 000g (12 000rpm) to dry the EZ-10 membrane. Discard flow-through and transfer the spin column to a clean 1.5ml centrifuge tube.
10. Add 50-100µl TE Buffer directly onto the center part of EZ-10 membrane. Incubate at room temperature for 1min and then centrifuge for 1min at 9 000g (12 000rpm) to elute the DNA.

Protocol n° 7. *Kit n°5.a* – DNeasy Plant Pro Kit – QIAGEN

1. Add 5-100mg of fresh or frozen plant tissue and 500µl of Solution CD1 to a 2ml tissue disruption tube. Vortex briefly to mix.

**2.** Homogenize: Secure tissue disruption tubes to a Vortex Adapter and vortex at maximum speed for 10min.<br>**3.** Centrifuge the tissue disruption tubes at 12 000g for 2min.<br>**4.** Transfer the supernatant to a clean 1.5ml microcentrifuge tube (provided).<br>**5.** Add 200µl Solution CD2 and vortex for 5s.<br>**6.**Centrifuge at 12 000g for 1min at room temperature. Avoiding the pellet, transfer the supernatant to a clean 1.5ml microcentrifuge tube (provided).<br>**7.** Add 500µl of Buffer APP and vortex for 5s.<br>**8.** Load 600µl lysate onto an MB Spin Column. Centrifuge at 12 000g for 1min.<br>**9.** Discard the flow-through and repeat step 8 to ensure that all the lysate has passed through the MB spin column.<br>**10.** Place the MB spin column into a clean 2 ml collection tube (provided).<br>**11.** Add 650µl Buffer AW1 to the MB spin column. Centrifuge at 12 000g for 1min. Discard the flow-through and place the MB spin column back into the same 2ml collection tube.<br>**12.** Add 650µl of Buffer AW2 to the MB spin column. Centrifuge at 12 000g for 1min. discard the flow-through and place the MB spin column into the same 2ml collection tube.<br>**13.** Centrifuge at up to 16 000g for 2min. Place the MB spin column into a new 1.5 ml elution tube (provided).<br>**14.** Add 50-100µl of buffer EB to the center of the white filter membrane.<br>**15.** Centrifuge at 12 000g for 1min. Discard the MB spin column. The DNA is now ready for downstream applications.
**Protocol n° 8.** *Kit n°5.b* – DNeasy Plant Pro Kit – QIAGEN<br><br>**1.** Add 5-100mg of fresh or frozen plant tissue and **450µl of Solution CD1 and 50µl** to a 2ml tissue disruption tube. Vortex briefly to mix.<br>**2.** Homogenize: Secure tissue disruption tubes to a Vortex Adapter and vortex at maximum speed for 10min.<br>**3.** Centrifuge the tissue disruption tubes at 12 000g for 2min.<br>**4.** Transfer the supernatant to a clean 1ml microcentrifuge tube (provided).<br>**5.** Add 200µl Solution CD2 and vortex for 5s.<br>**6.**Centrifuge at 12 000g for 1min at room temperature. Avoiding the pellet, transfer the supernatant to a clean 1.5ml microcentrifuge tube (provided).<br>**7.** Add 500µl of Buffer APP and vortex for 5s.<br>**8.** Load 600µl lysate onto an MB Spin Column. Centrifuge at 12 000g for 1min.<br>**9.** Discard the flow-through and repeat step 8 to ensure that all the lysate has passed through the MB spin column.<br>**10.** Place the MB spin column into a clean 2 ml collection tube (provided).<br>**11.** Add 650µl Buffer AW1 to the MB spin column. Centrifuge at 12 000g for 1min. Discard the flow-through and place the MB spin column back into the same 2ml collection tube.<br>**12.** Add 650µl of Buffer AW2 to the MB spin column. Centrifuge at 12 000g for 1min. discard the flow-through and place the MB spin column into the same 2ml collection tube.<br>**13.** Centrifuge at up to 16 000g for 2min. Place the MB spin column into a new 1.5ml elution tube (provided).<br>**14.** Add 50-100µl of buffer EB to the center of the white filter membrane.<br>**15.** Centrifuge at 12 000g for 1min. Discard the MB spin column. The DNA is now ready for downstream applications.

Protocol n° 9. *Kit n°5.c* – Pre-Optimized DNeasy Plant Pro Kit – QIAGEN

1. Add 5-100mg of fungi/mushroom tissue (treated as described in correspond part of the manuscript) and 500µl of Solution CD1 to a 2ml tissue disruption tube. Vortex briefly to mix.
2. Homogenize: Secure tissue disruption tubes to a Vortex Adapter and vortex at maximum speed for 10 min.
3. Centrifuge the tissue disruption tubes at 12 000g for 2min.
4. Transfer **exactly 400µl** of the supernatant to a clean and **autoclaved** 1.5ml microcentrifuge tube.
5. Add 200µl Solution CD2 and vortex for 5s.
6.Centrifuge at 12 000g for 1min at room temperature. Avoiding the pellet, transfer **exactly 450µl** of the supernatant to a clean and **autoclaved** 1.5ml microcentrifuge tube.
7. Add 500µl of Buffer APP and vortex for 5 s.
8. Load 600µl lysate onto an MB Spin Column. Centrifuge at 12 000g for 1min.
9. Discard the flow-through and repeat step 8 to ensure that all the lysate has passed through the MB spin column.
10. Centrifuge at 12 000g for 1min.
11. Place the MB spin column into a clean and **autoclaved** 2ml collection tube.
12. Add 650µl Buffer AW1 to the MB spin column. Centrifuge at 12 000g for 1min. Discard the flow-through and place the MB spin column back into the same 2ml collection tube.
13. Centrifuge at 12 000g for 1min.
14. Add 650µl of Buffer AW2 to the MB spin column. Centrifuge at 12 000g for 1min. discard the flow-through and place the MB spin column into the same 2ml collection tube.
15. Centrifuge at 15 000g for 4min.
16. Place the MB spin column into a new, clean and autoclaved 1.5ml microcentrifuge tube.
17. Add **15-50**µl of buffer EB to the center of the white filter membrane. **Incubate for 30s-2min of contact**.
18. Centrifuge at 13 000g for 2min.
19. Store the used tissue disruption tube with its pellet and the MB spin column at -20°C, if the operator estimated another extraction round for the same variety.
20. The DNA is now ready for downstream quantification, qualification and sequencing analyses. Nevertheless, gDNA should be stored at -20°C. When needed, it should be adapted for 12-24h at 4°C before daily use. If gDNA volume is important, aliquot it and store the stock at -80°C.

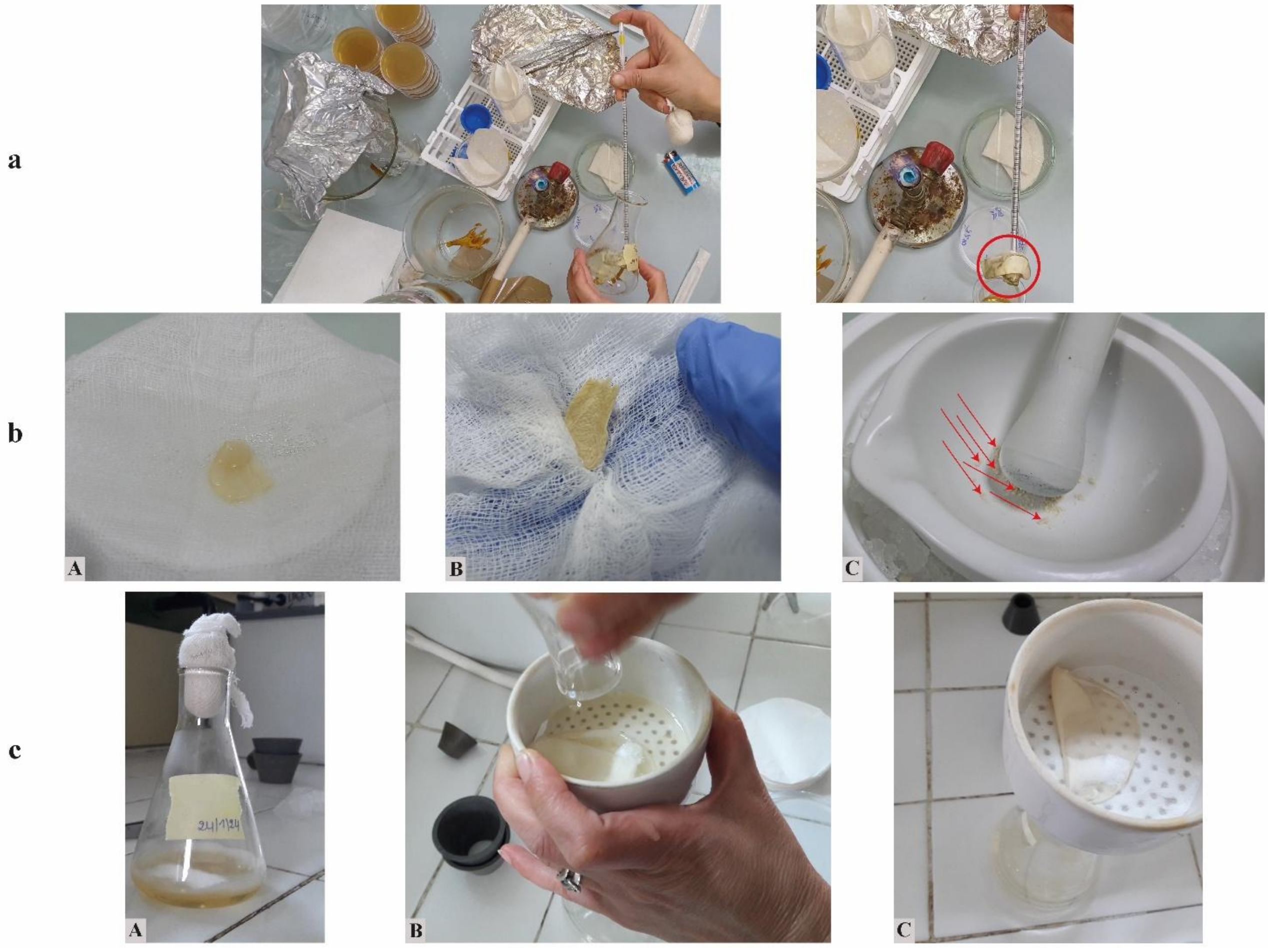

a: Preparation A
b: Preparation C (A.Fresh, Rinsed & B. Squeezed) and Preparation D (A.Fresh & Rinsed, B.Squeezed & C.Fine powder)
c: Preparation B

Supplemental Figure S1. Sample preparation methods

Supplemental Table S2. Used GenBank accession number sequences in molecular phylogenetic analysis

The bold accession number sequences are newly generated and submitted for this study.

Sequences	Culture Collection Designation	*ITS* Accession Number
**ITS Region**	**BS6**	**PV635458**
	Clitopilus sp. NWFVA5901	MZ045858.1
	Clitopilus baronii FDS-CA-05337	PQ361878.1
	Clitopilus baronii AMB 18357	MN855363.1
	Clitopilus baronii AMB 18362	MN855368.1
	Clitopilus baronii K (M) 179703	MN855362.1
	Cliptopilus baronii CA FUNDIS iNaturalist #164651291	OR771818.1
	Cliptopilus baronii HAY-F006132	PP335778.1
	**BS200**	**PV629228**
	Porostereum spadiceum	OW988257.1
	Porostereum spadiceum Psp23-22A	OP718302.1
	Porostereum spadiceum MUT<ITA>:2492	MF140468.1
	Porostereum spadiceum	OW988254.1
	Polyporales sp. 1 SR-2012 strain 1165	JQ312137.1
	Porostereum spadiceum Ps28C-21A	OK614921.1
	Ceriporiopsis kunmingensis Cdh1	PQ452406.1
	Porostereum spadiceum LZ11-10	MT898680.1
	**BS22-9**	**PV635497**
	Trametes versicolor ICMP:19973	KF727428.1
	Trametes versicolor TRAMS	MK509800.1
	Trametes versicolor 170216GAR16P5	MW826127.1
	Trametes versicolor 5H1_P0_P5_2	KM232462.1
	Trametes versicolor 679J	PP523907.1
	Trametes versicolor HHB12282 *sp*	JN164974.1
	Phellinus igniarius wb434	AF455434.1
	**BS22-13**	**PV635514**
	Schizophyllum commune NAAS02317	MG664217.1
	Schizophyllum commune Sc26M-21A	OK647409.1
	Schizophyllum commune CNM-CM8857	MN313584.1
	Chondrostereum purpureum GQD-7-1	MN626467.1
	**BS23-14**	**PV635498**
	Gloeophyllum abietinum H 22988	JX524619.1
	Gloeophyllum abietinum 254	AJ420947.1
	Gloeophyllum abietinum 256	AJ420948.1
	Gloeophyllum abietinum DSM 1210	GQ354271.1
	Gloeophyllum abietinum Dai_22862	OL457965.1
	Gloeophyllum abietinum 10476	JX524621.1
	Gloeophyllum striatum 0904/72-J	KC345720.1
	Gloeophyllum striatum 1624	KC345723.1

	Gloeophyllum sepiarium 3555	MK268936.1
	Gloeophyllum sepiarium 3559	MK268937.1
	Gloeophyllum subferrugineum FRI417R	KJ141173.1
	**BS100**	**PV635190**
	Irpex laceratus G41	MH880256.1
	Irpex laceratus CREA-DC TPR OL.112	MW677465.1
	Polyporales sp. 1 454B	JQ312153.1
	Polyporales sp. 1 718	JQ312208.1
	Irpex laceratus CS3_15	MK460901.1
	Irpex laceratus CREA-DC TPR OL.119	MW677474.1
	Irpex rosettiformis F25-02	KX664355.1
	**GC9**	**PV635457**
	Trichoderma asperellum THP2	MW074098.1
	Metarhizium rileyi TAM3	MG893852.1
	Trichoderma asperellum TF1	KU341007.1
	Metarhizium rileyi TAM2	MG893851.1
	Trichoderma asperellum T337	KP059114.1
	Trichoderma asperellum TRB1	OP363989.1
	Trichoderma asperellum AUMC 16439	PP990201.1
	Trichoderma asperellum D4	MG198706.1
	**S3**	**PV635456**
	Trichoderma harzianum PAN12-18	MK322679.1
	Trichoderma harzianum VRU-Th108	KJ000324.1
	Trichoderma harzianum PAN12-66	MK322681.1
	Trichoderma harzianum FU-EFA 767.17	PP661033.1
	Hypocrea sp. ZG16	JQ775560.1
	Trichoderma simmonsii UTFC 10063	MG132084.1
	Hypocrea lixii C.P.K. 1936	FJ860767.1
D1/D2 domain	**BS6**	**PV636529**
	Clitopilus sp. DSM100324_C28_RLCS17	MT453280.1
	Clitopilus abprunulus F-529	PQ653177.1
	Clitopilus prunulus GLM 45889	AY207161.1
	Uncultured *Basidiomycota* clone 4s1_e04	EU489989.1
	Clitopilus baronii F-2393	PQ638969.1
	Clitopilus baronii F-835	PQ652399.1
	Clitopilus baronii F-2407	PQ652714.1
	Clitopilus pinsitus	GQ289148.1
	Clitopilus hobsonii QYL-10	OK655769.1
	**BS200**	**PV636977**
	Porostereum spadiceum Wu 9708-104	DQ679918.1
	Porostereum spadiceum Wu 9508-139	MZ637263.1
	Porostereum spadiceum KHL 13438	OR822131.1
	Porostereum umbrinoalutaceum NLB 1261	ON715807.1
	Porostereum fulvum LY:18491	MG649454.1

	Ceriporiopsis carnegieae CFMR:ERC-71-366-R	OL376623.1
	Ceriporiopsis carnegieae RLG7277T	KY948854.1
	**BS22-9**	**PV636530**
	Trametes versicolor X-12	KC176313.1
	Trametes versicolor C-4	DQ208417.1
	Trametes versicolor YG-J3	MT524624.1
	Trametes versicolor KMRB18111321	ON402848.1
	Trametes versicolor isolate wood 3-4	KC176304.1
	Trametes ochracea CBS:289.33	MH866894.1
	Vanderbylia delavayi 7182	KX880694.1
	Trametes pubescens CBS:367.34	MH867075.1
	**BS23-13**	**PV636531**
	Schizophyllum commune IFM 62603	LC798818.1
	Schizophyllum commune CBS:405.96	MH874209.1
	Uncultured fungus OTUL1	KR905953.1
	Schizophyllum commune NLB 1317	ON715808.1
	Schizophyllum sp. PDD 103380	KF727350.1
	Schizophyllum radiatum UTHSCDI14-2	LT217572.1
	**BS23-14**	**PV636532**
	Gloeophyllum abietinum P254	AJ583431.1
	Gloeophyllum abietinum Dai_22862	OL457435.1
	Gloeophyllum abietinum H 22988	KC782733.1
	Gloeophyllum sepiarium CBS:317.50	MH868155.1
	Gloeophyllum sepiarium CFMR Wilcox-3BB	NG_060630.1
	Gloeophyllum subferrugineum FRI417R	KJ141184.1
	Gloeophyllum subferrugineum 1218	KC782738.1
	Gloeophyllum trabeum FP101508	KJ141187.1
	Heliocybe sulcata OMC1185	OL898491.1
	Neolentinus kauffmanii CBS:315.50	MH868153.1
	**BS100**	**PV636976**
	Irpex latemarginatus MIT1/3	PV030202.1
	Irpex laceratus CREA-DC	MW714619.1
	Irpex laceratus CREA-DC TPR OL.109	MW714615.1
	Irpex lacerates	MK447560.1
	Irpex latemarginatus CBS:436.48	MH867973.1
	Irpex laceratus CREA-DC TPR OL.124	MW714620.1
	Irpex laceratus CREA-DC TPR OL.128	MW714622.1
	Hexagonia hydnoides CBS:360.34	MH867071.1
	Ceriporia sp. OTU68 TS-2013	AB808462.1
	Ceriporia lacerata Dai10734	JX644068.1
	**GC9**	**PV636528**
	Trichoderma asperelloides SZMC 27998	PP336494.1
	Trichoderma asperellum IC01	ON054057.1

	Trichoderma asperellum CEN768	OM515036.1
	Trichoderma viride	AY291123.1
	Trichoderma hamatum CEN1350	OM515061.1
	Trichoderma paucisporum CBS 118645	NG_069881.1
	Trichoderma asperellum APVR 24	KY643783.1
	Trichoderma asperellum JX14A04	PP381152.1
	Trichoderma asperellum S1-26-F	MG675227.1
	**S3**	**PV636533**
	Trichoderma harzianum NS3	OQ552851.1
	Trichoderma atrobrunneum AH07E02	PP380705.1
	Trichoderma harzianum ICO2	ON054038.1
	Trichoderma afroharzianum CEN158	OM515012.1
	Trichoderma atrobrunneum SD17A03	PP381462.1
	Trichoderma harzianum CBS:130686	MH877289.1
	Trichoderma harzianum UNIJAG.PL.OP981	ON193839.1
	Trichoderma harzianum ERVR07	MK182367.1
**Combined ITS+D1/D2**	**BS6**	----------------
	Clitopilus baronii isolate F-2407	PQ652714.1
	Clitopilus baronii isolate F-2393	PQ638969.1
	Clitopilus baronii isolate F-835	PQ652399.1
	Clitopilus hobsonii strain QYL-10	OK655769.1
	**BS200**	----------------
	Bjerkandera mikrofumosa voucher MV353	MH025416.1
	Porostereum spadiceum voucher KHL 13438	OR822131.1
	Polyporales sp. 1 SR-2012 strain 1165	JQ312137.1
	Porostereum umbrinoalutaceum strain N.L. Bougher NLB 1261	ON715807.1
	Porostereum spadiceum isolate Psp23-22A	OP718302.1
	Porostereum spadiceum	OW988257.1
	**BS22-9**	
	Vanderbylia delavayi voucher Dai 7182	KX880694.1
	Trametes conchifer IFM 65269	LC798846.1
	Trametes versicolor isolate X-29 haplotype b	KC176325.1
	Trametes versicolor isolate X-12	KC176313.1
	Trametes versicolor isolate X-29 haplotype a	KC176324.1
	Trametes versicolor isolate T-868	KC176302.1
	**BS23-13**	----------------
	Schizophyllum commune strain LP02	KX034183.1
	Schizophyllum commune strain 207.1	PP336468.1
	Schizophyllum commune IFM 65574	LC798860.1
	Schizophyllum commune IFM 65403	LC798853.1
	Schizophyllum commune CBS 199.27	LC798929.1
	Schizophyllum commune IFM 62604	LC798819.1
	**BS23-14**	----------------

	Gloeophyllum sepiarium voucher Mushroom Observer 428410	MZ354634.1
	Gloeophyllum sepiarium voucher Mushroom Observer 426375	MW633056.1
	Gloeophyllum sepiarium voucher OM12420	JN649344.1
	Gloeophyllum trabeum culture CFMR:617	MN783219.1
	Gloeophyllum sepiarium voucher Mushroom Observer 426375	MW633056.1
	Gloeophyllum abietinum voucher H 22988	JX524619.1
	Gloeophyllum abietinum isolate 256 (G 334)	AJ420948.1
	Gloeophyllum abietinum isolate 254 (G 227)	AJ420947.1
	Gloeophyllum abietinum strain DSM 1210	GQ354271.1
	Gloeophyllum abietinum voucher Dai 3595	JX524620.1
	Gloeophyllum abietinum strain 484	KM098122.1
	Gloeophyllum abietinum voucher Cui 10476	JX524621.1
	Gloeophyllum abietinum culture-collection MUT<ITA_:5488	KM264291.1
	**BS100**	----------------
	Ceriporiopsis resinascens voucher F20091106KCM38	GU080235.1
	Irpex laceratus isolate G41	MH880256.1
	Irpex latemarginatus voucher MO317736	MT551951.1
	Irpex rosettiformis isolate F25-02	KX664355.1
	Polyporales sp. 1 SR-2012 strain 454B	JQ312153.1
	Ceriporia sp. 1 RG-2014 strain MX106	KJ831954.1
	Irpex rosettiformis isolate F24-03	KX664351.1
	Irpex rosettiformis isolate F27-05	KX664366.1
	Irpex latemarginatus voucher Marcin Piatek 4.IX.1997 (H)	KX752592.1
	Lentinus cartilagineus isolate 29R-4-F03	KX958085.1
	**GC9**	----------------
	Trichoderma sp. CHTAM24	JF773648.1
	Fusarium lateritium strain SGLMf40	EU715687.1
	Trichoderma asperellum strain APVR 07	KY643785.1
	Trichoderma asperellum	MH260587.1
	Trichoderma asperellum isolate J111	OQ538316.1
	Trichoderma asperellum strain ZJ11D06	PP381708.1
	**S3**	----------------
	Hypocrea pilulifera strain SGLMf38	EU715685.1
	Nectria pseudotrichia strain SGLAf45	EU715616.1
	Fusarium lateritium strain SGSGf33	GQ377491.1
	Trichoderma harzianum isolate CTCCSJ-F-KZ40809	KY750444.1
	Trichoderma harzianum strain TH XV	MH602422.1
	Trichoderma harzianum strain TH X	MH602417.1

	Trichoderma harzianum isolate 8227	KF454872.1
	Trichoderma harzianum isolate PAN12-74	MK322682.1
	Trichoderma harzianum isolate Ting Ho 2	OR091357.1
	Trichoderma harzianum isolate PAN12-50	MK322680.1
**Outgroups**	*Neurospora crassa* CBS:709.71	MH860307.1
	Lentinula edodes M.A.S.R	PP498976.1
	Pleurotus ostreatus CBS:291.47	MH856257.1
	Agaricus bisporus CBS:116.68	MH859080.1

Supplemental Table S3. Applied NGS technologies during this study on the validated gDNA fungal samples

Samples	***Kits***	***Protocols n°***	***Types***	***gDNA Assessment Workflow***			***Illumina Technologies***				***Oxford Nanopore Technology***			
				NanoDrop	Qubit	Electrophoresis	Yes/No	Library	Device	Where?	Yes/No	Library	Device	Where?
BS23-14	1	1	*B*	√	√	√	Y	Nextera XT DNA Library Prep (August 2021)	MiSeq	CBS	N	None		
BS6	5	7	*B*	√	√	√	N	None			Y	Rapid Barcoding Kit 96 V14	MinION Flow Cell R10.4.1	CBS
BS6	5	9	*C*	√	√	√								
BS6	5	9	*C*	√	√	√	Y	Illumina DNA Prep	NestSeq550	HCN	N	None		
BS200	5		*C*	√	√	√								
BS22-9	5		*C*	√	√	√								
BS22-13	5		*C*	√	√	√								
S3	5		*C*	√	√	√								
GC9	5		*C*	√	√	√								
BS100	5	Optimized	*D*	√	√	√								
BS23-14	5		*D*	√	√	√								

CBS: Centre of Biotechnology of Sfax, Tunisia
HCN: Hôpital Charles Nicolle de Tunis, Tunisia

Supplemental Table S4. Tools used in the bioinformatics analyses

Tools (version) — Platform / File	*MiSeq*[1] *Scaffolds*	*NextSeq550*[2] *Scaffolds*	*MinION Mk1C*[3] *Consensus*
Trimmomatic (v.0.39)[a]	√	√	-
Porechop (v.0.2.4)[b]	-	-	√
SPAdes (v.3.15.4)[c]	√	√	-
Flye (v.2.9.1)[d]	-	-	√
QUAST (v.5.2.0)[e]	√	√	√
gfastats (v.1.3.6)[f]	√	√	√
BandageInfo (*v2022.09*)[g]	√	√	√
BUSCO "Metaeuk" (*v.5.4.6*)[h]	√	√	√

[a]Parameters: pair_of_files; ILLUMINACLIP: yes, Custom adapter sequences in fasta format: DNA_Library_Prep CTGTCTCTTATACACATCT; Maximum mismatch: 2; accurate the match between the two 'adapter ligated':30; accurate the match between any adapter:10; minimum length of adapter that needs to be detected: 8; always keep both reads:True; Select Trimmomatic operation to perform: SLIDINGWINDOW; Number of bases to average across:5; Average quality required:25; Select Trimmomatic operation to perform: MILEN; Minimum length of reads to be kept: 50; Quality score encoding: Nothing selected

-threads 1 fastq_r1.fastqsanger.gz fastq_r2.fastqsanger.gz fastq_out_r1_paired.fastqsanger.gz fastq_out_r1_unpaired.fastqsanger.gz fastq_out_r2.fastqsanger.gz fastq_out_r2_unpaired.fastqsanger.gz ILLUMINACLIP:/home/galaxy/galaxy/database/jobs_directory/000/458/configs/tmpdxa_4bs2:2:30:10:8:true SLIDWINDOW:5:25 MINLEN:50 -trimlog trimlog

[b]Parameters: Galaxy Default Parameters

[c]Parameters: Galaxy Default Parameters for paired-end short-reads

With the followed System Information:
OS: Linux-6.5.0-21-generic-x86_64-with-glibc2.35
Python version: 3.9.0
Memory limit: 8Gb

[d]Parameters: Galaxy Default Parameters

[e,1,3&4]Parameters: Assembly mode: co; contigs/scaffolds file: consensus; type of assembly: genome; use a reference genome: false; type of organism: Eukaryote: use of GeneMark-ES for gene finding, Barnap for ribosomal RNA genes prediction, BUSCO for conserved orthologs finding (--eukaryote); minimum IDY% considered as proper alignment: 95.0; lower threshold for a contig length (in bp): 500

consensus -o outputdir --eukaryote --min-identity 95.0 --min-contig 500 --split-scaffolds --min-alignment 65 --ambiguity-usage one --ambiguity-score 0.99 --local-mis-size 200 --contig-thresholds 0,1000 --extensive-mis-size 1000 --scaffold-gap-max-size 1000 --unaligned-part-size 500 --x-for-Nx 90

With the followed System Information:
OS: Linux-6.8.0-40-generic-x86_64-with-glibc2.35 (linux_64)
Python version: 3.10.13
CPUs number: 4

[e,2]Parameters: Assembly mode: co; contigs/scaffolds file: Scaffolds; type of assembly: genome; use a reference genome: false; type of organism: Eukaryote: use of GeneMark-ES for gene finding, Barnap for ribosomal RNA genes prediction, BUSCO for conserved orthologs finding (--eukaryote); minimum IDY% considered as proper alignment: 95.0; lower threshold for a contig length (in bp): 500

scaffolds -o outputdir --eukaryote --min-identity 95.0 --min-contig 500 --split-scaffolds --min-alignment 65 --ambiguity-usage one --ambiguity-score 0.99 --local-mis-size 200 --contig-thresholds 0,1000 --extensive-mis-size 1000 --scaffold-gap-max-size 1000 --unaligned-part-size 500 --x-for-Nx 90

With the followed System Information:
OS: Linux-6.5.0-21-generic-x86_64-with-glibc2.35 (linux_64)
Python version: 3.10.13
CPUs number: 4

[f,1&3]Parameters: Input_file: Scaffolds; Specify target sequences: false, tool mode: statistics; report mode: assembly; thousands separator in output: true; tabular-format output: true;500 generates the initial set of paths: false

[f,2]Parameters: Input_file: Scaffolds; Specify target sequences: false, tool mode: statistics; report mode: assembly; thousands separator in output: true; tabular-format output: true;500 generates the initial set of paths: false

[f,4]Parameters: Input_file: consensus; Specify tar5get sequences: false, tool mode: statistics; report mode: assembly; thousands separator in output: true; tabular-format output: true;500 generates the initial set of paths: false

[h]Parameters: Default parameters

[i]Parameters: First run with Ascomycota as a select-lineage | Second run with Basidiomycota as a selected lineage. All the three runs were lunched with galaxy default parameters which are mainly Mode: euk_genome_met, *Metaeuk* as gene predictor: no, E-value cutoff for BLAST searches: 0.001, How many candidate regions to consider: 3

Supplemental Table S5. List of nucleotide sequence and whole genome sequence accession numbers of the 8 Tunisian fungal strains

Strains	BioSample	TaxID	ITS	D1/D2	WGS	Sequencing platform
BS6	SAMN46403166	2690848	PV635458	PV636529	SRR32113025<br>SRR32132663[a]<br>SRR32132728[b]	NextSeq550<br>MinION Mk1C-1st essay<br>MinION Mk1C-2nd essay
BS200	SAMN48330156	103378	PV629228	PV636977	SRR33421921[d]	NextSeq550
BS22-9	SAMN46416581	5325	PV635497	PV636530	SRR32126389[d]	
BS22-13	SAMN46409157	5334	PV635514	PV636531	SRR32118528[d]	
BS23-14	SAMN46537950	180171	PV635498	PV636532	SRR32220537[d]<br>SRR32248698[c]	
BS100	SAMN48330131	194684	PV635190	PV636976	SRR33421360[d]	
GC9	SAMN46419714	101201	PV635457	PV636528	SRR32129886[d]	
S3	SAMN46545462	5544	PV635456	PV636533	SRR32227576[d]	

Supplemental Table S6. gDNA concentration and purity obtained from different applied protocols

Samples	Protocol Number	Preparation Type	Mycelia Yield (*g*)	*Nanodrop2000*			*Qubit.3* (*ng/µl*)
				[*ng/µl*]	$A_{260/280}$	$A_{260/230}$	*HS*
**S6.a. Comparison between the applied protocols**							
**BS22-9**	*1*	B	≈1	225.4	2.06	1.83	83.4
**BS23-14 (diluted ½)**				220.7	1.87	0.89	97.6
**BS200**	*2*	B	≈0.2	29.7	1.86	0.5	OR_L
**BS200**	*3*	B	0.5	60.2	2.14	0.67	3.74
**BS200**	*4*	B	0.5	553.9	2.25	1.40	84
**BS6**			0.429	513.9	2.19	2.46	100
**BS200** – *Rep1*	5	B	≈0.1	1.4	-9	4	n.d
**BS200** – *Rep 2*				1.3	51	1.56	n.d
**BS200** – *Rep1*	*6*	B	≈0.1	0.8	1.08	0.02	n.d
**BS200** – *Rep 2*				7.1	1.54	0.13	n.d
**BS6**	*7*	B	0.156	63.5	1.89	1.7	60
**BS200**			0.297	62.3	1.94	1.80	49
**S6.b. DNeasy Plant Pro Kit (Kit 5) with manufacturer's instructions with or without PS solution**							
**BS200**	*7*	B	≈0.1	34.2	1.79	1.03	28.8
**BS200**	8			35.4	1.82	0.06	28
**S6.c. Verification**							
**BS6** – *Rep1*	*7*	B	0.125	125	1.92	0.57	OR_H
**BS6** – *Rep2*			0.110	129.8	2.01	0.17	OR_H
**BS6** – *Rep3*			0.117	66.6	1.9	0.44	61.4
**BS6** – *Rep4*			0.139	67.8	1.89	0.79	67.4
**BS200** – *Rep1*	*7*	B	0.178	23.5	2.05	0.60	21.6
**BS200** – *Rep2*			0.192	26.3	2.02	1.66	27.4
**BS200** – *Rep3*			0.194	38.2	1.98	2.98	39
**BS200** – *Rep4*			0.189	21.9	2.01	1.75	21.8
**S6.d. Pre-optimized protocol: Sterile fresh directly extracted VS Fresh, rinsed, squeezed, and frozen Mycelia at -20°C**							
**BS200** – *Rep1*	*9*	A	0.129	48.5	1.85	1.59	1.55
**BS200** – *Rep2*			0.072	48.4	1.87	0.63	2.96
**BS100** – *Rep1*			0.077	11.7	1.49	0.71	1.10
**BS100** – *Rep2*			0.1	18.1	1.78	1.08	2.40
**GC9** – *Rep1*			0.086	35.3	1.78	0.75	2.64
**GC9** – *Rep2*			0.086	17.9	1.60	0.74	1.66
**S3** – *Rep1*			0.076	25	1.72	0.53	1.44
**S3** – *Rep2*			0.040	18.1	1.58	0.16	0.077
**BS22-9** – *Rep1*			0.255	24.2	-3	1.25	3.62
**BS222-9** – *Rep2*			0.255	51	7.05	1.25	7.94
**BS6** – *Rep1*			0.415	63.7	4.52	1.01	8.52

**BS6** – *Rep2*			0.428	81.1	3.28	0.7	12
**BS22-13**– *Rep1*			0.335	-4.3	0.19	-0.05	OR_L
**BS22-13** – *Rep2*			0.335	-7.4	0.30	-0.07	0.118
**BS6** – *Rep1*			0.151	369.3	1.88	1.78	338*
**BS6** – *Rep2*			0.151	119.5	1.85	1.07	131*
**BS200** – *Rep1*			0.106	278.2	1.89	1.77	220*
**BS200** – *Rep2*			0.106	201.9	1.87	1.24	248*
**BS22-9** – *Rep1*			0.151	219.6	1.87	1.96	232*
**BS222-9** – *Rep2*			0.151	187.4	1.84	1.58	246*
**BS22-13**– *Rep1*	*9*	C	0.152	9.4	1.88	0.67	8.92*
**BS22-13** – *Rep2*			0.152	12.1	1.26	0.11	14.3*
**BS100** – *Rep1*			0.177	45.9	1.78	1.33	46.2*
**BS100** – *Rep2*			0.177	56.0	1.85	1.38	57*
**S3** – *Rep1*			0.228	95.0	1.83	0.63	106*
**S3** – *Rep2*			0.228	90.2	1.82	1.65	88*
**GC9** – *Rep1*			0.185	172.1	1.89	0.51	147*
**GC9** – *Rep2*			0.185	127.1	1.88	1.98	110*
**BS22-13**			0.023	11.8	1.96	0.10	9.52*
**BS100**			0.14	213.4	1.86	1.72	162*
**S3**	*9*	D	0.032	52.3	1.72	0.24	45.8*
**GC9**			0.267	385.7	1.92	1.23	398*
**BS23-14**			0.159	69.1	1.82	1.84	90.8*
**S6.e. Validation – Optimized protocol**							
**BS200**			0.391	79.6	1.89	1.86	74.2*
**BS6**			0.470	173.8	1.91	0.70	175*
**BS22-9**			0.481	446.3	1.90	0.54	444*
**BS22-13**			0.485	28.7	1.97	0.57	20.2*
**BS23-14**	*10*	D	0.470	214.7	1.92	1.91	200*
**BS100**			0.404	203.8	1.92	2.25	206*
**GC9**			0.391	23.6	1.78	1.07	10.1*
**S3**			0.405	180.3	1.92	0.98	164*

OR: Out of Range – L: Low – H: High – n.d : Not determined
*Qubit.3 with Br manufacturer's instructions
Preparation A: Fresh mycelium; **Preparation B**: Mycelium filtered with a non-sterile filtering system; **Preparation C**: Fresh, rinsed, squeezed, and frozen; **Preparation D**: Cryogenic Treated mycelium after being prepared as C.

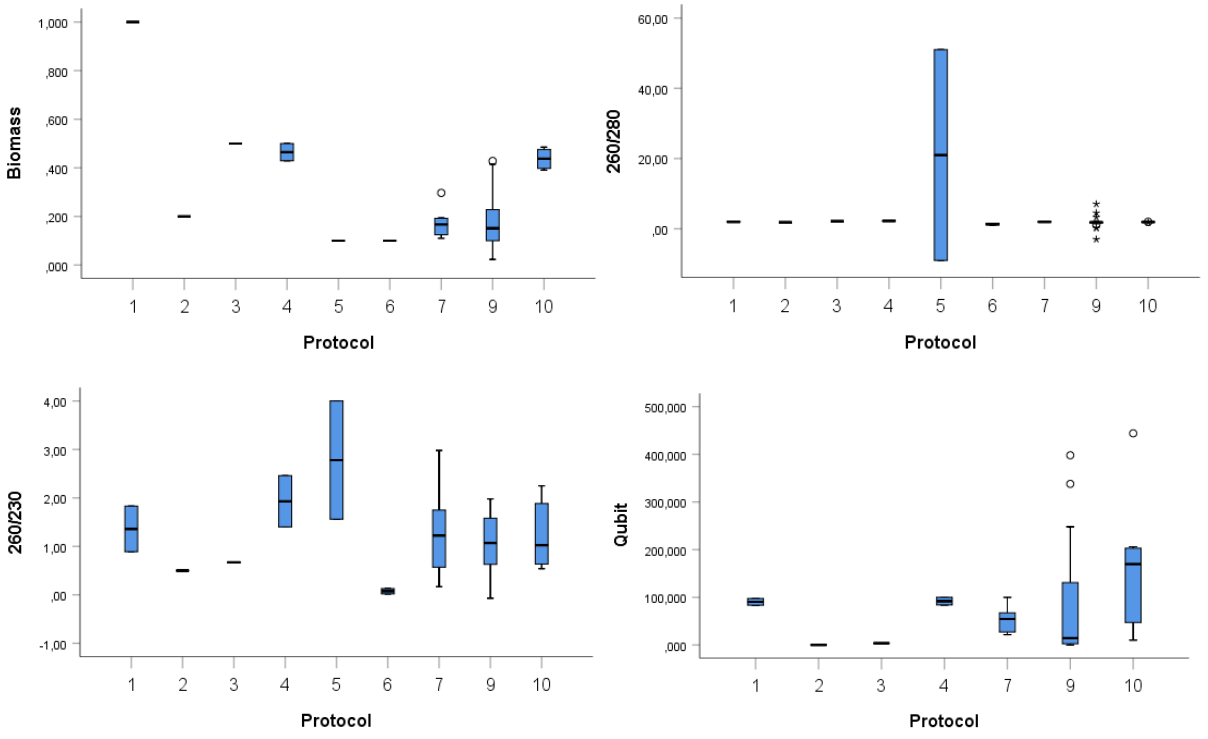

Supplemental Figure S2. Comparison between different applied protocols in terms of biomass, purity (260/280 & 260/230) and yield (qubit) in the first phase (see Table S6.a)

Supplemental Table S7. BLAST algorithm detailed report Molecular identification of newly isolated medical mushrooms by ITS1-5.8S-ITS2 and D/D2 region of rRNA gene sequence analysis

			Studied Sequences			Closet relative	
**N°**	**Samples**	**Barcodes**	Length (bp)		Sequences GenBank accession no.	Closest relative (latin name, Cover, Identity)	GenBank accession no.
			Each	*Combined*			
**1**	*BS6*	ITS1-5.8S-ITS2	597	1,198	*PV635458*	(*Clitopilus baronii*, 88%, 91.42%)	*MN855363.1*
		D1/D2	**601**		*PV636529*	(*Clitopilus baronii*, 91%, 97.64%)	*PQ652714.1*
**2**	*BS200*	ITS1-5.8S-ITS2	**606**	**1,207**	*PV629228*	(*Porostereum spadiceum*, 99%, 99.17%)	*OR822131.1*
		D1/D2	601		*PV636977*	(*Porostereum spadiceum*, 99%, 96.98%)	*OR822131.1*
**3**	*BS22-9*	ITS1-5.8S-ITS2	572	1,150	*PV635497*	(*Trametes versicolor*, 100%, 98.78%)	*PV109040.1*
		D1/D2	578		*PV636530*	(*Trametes versicolor*, 99%, 99.30%)	*AM269878.1*
**4**	*BS23-13*	ITS1-5.8S-ITS2	587	1,184	*PV635514*	(*Schizophyllum commune*, 100%, 99.32%)	*OR062411.1*
		D1/D2	597		*PV636531*	(*Schizophyllum commune*, 99%, 99.66%)	*LC798819.1*
**5**	*BS23-14*	ITS1-5.8S-ITS2	584	**1,138**	*PV635498*	(*Gloeophyllum abietinum*, 100%, 95.89%)	*JX524619.1*
		D1/D2	**554**		*PV636532*	(*Gloeophyllum abietinum*, 98%, 91.04%)	*AJ583431.1*
**6**	*BS100*	ITS1-5.8S-ITS2	595	1,168	*PV635190*	(*Irpex latemarginatus*, 100%, 99.33%)	*OP749582.1*
		D1/D2	574		*PV636976*	(*Irpex latemarginatus*, 100%, 97.39%)	*PV030202.1*
**7**	*GC9*	ITS1-5.8S-ITS2	**561**	1,118	*PV635457*	(*Trichoderma asperellum*, 98%, 99.28%)	*MW074098.1*
		D1/D2	557		*PV636528*	(*Trichoderma asperellum*, 99%, 99.28%)	*PV081134.1*
**8**	*S3*	ITS1-5.8S-ITS2	575	1,135	*PV635456*	(*Trichoderma harzianum*, 98%, 99.30%)	*PV156891.1*
		D1/D2	560		*PV636533*	(*Trichoderma harzianum*, 100%, 99.29%)	*OQ552851.1*

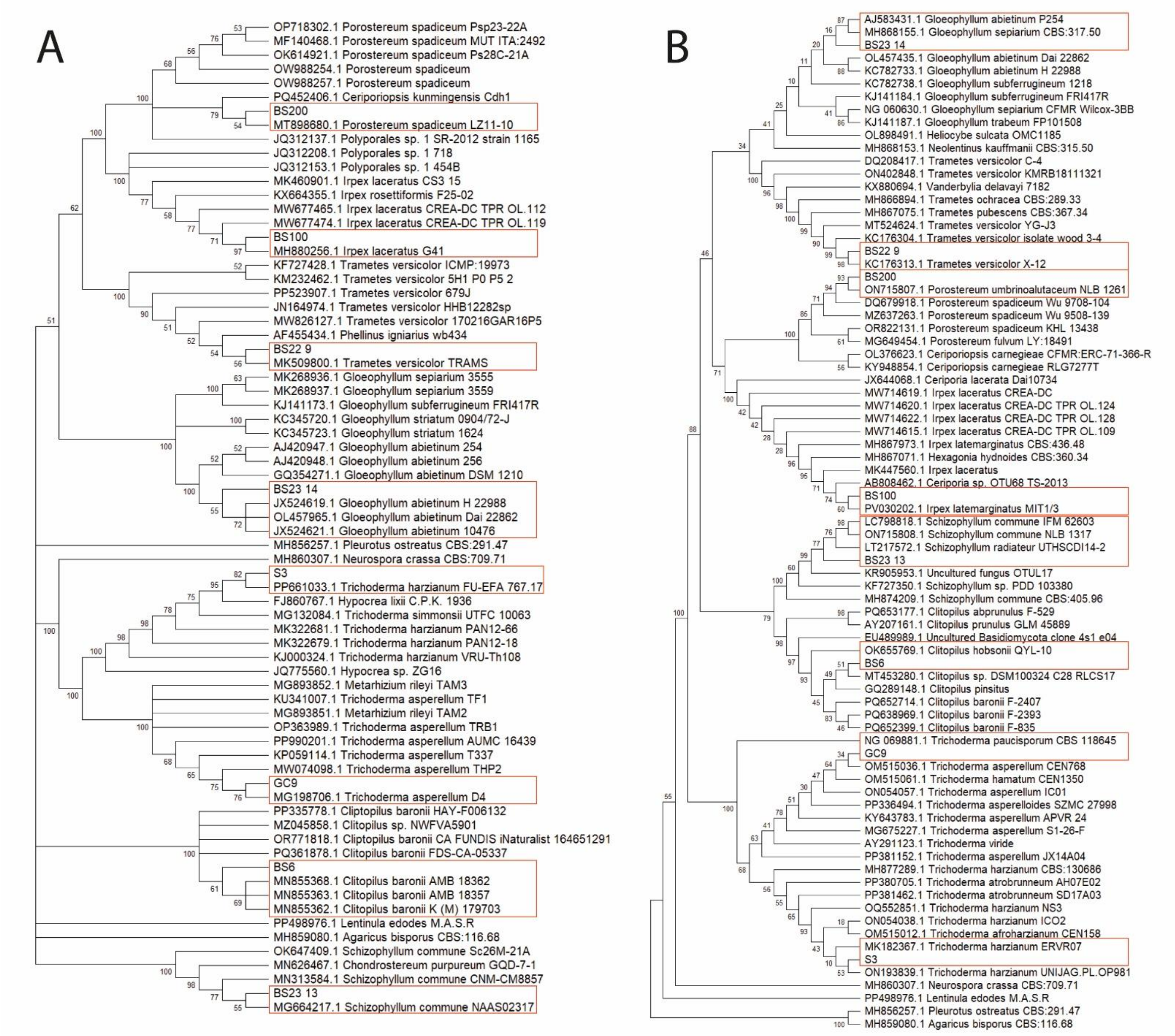

A : A phylogenetic tree based on ITS rDNA B : A phylogenetic tree based on D1/D2 rDNA domain

Sequences compared to reference sequences from Genbank showing the relationship of different strains to their closest strains

Supplemental Figure S3. ITS and D1D2 Phylogenetic Trees

Supplemental Table S8. gDNA concentrations, purity ratios and yield in each chosen sample for sequencing

Strains	Protocol Number	Preparation Type	Yield (*g*)	Nanodrop			Qubit (*ng/µl*)	Volume (*µl*)	gDNA yield (*µg*)	Sequencing platforms		
				[*ng/µl*]	$A_{260/280}$	$A_{260/230}$	*BR*			*Miseq* Nextera XT	*Mk1C* Rapid Barcoding	*Nextseq550* DNAprep
**BS23-14** (diluted 1/2)	1	B	≈1	210.7	2.12	2.14	36.4	50	1 820	√		
**BS6**	7	B	0.156	62.7	1.84	1.61	41	50	2 050		√	
**BS6**	9	C	0.151	369.3	1.88	1.78	338	35	11 830		√	√
**BS200**	9	C	0.106	278.2	1.89	1.77	220		7 700			√
**BS22-9**	9	C	0.151	219.6	1.87	1.96	232		8 120			√
**BS22-13**	9	C	0.152	9.4	1.88	0.67	8.92		312.2			√
**S3**	9	C	0.228	95.0	1.83	0.63	106		3 710			√
**GC9**	9	C	0.185	172.1	1.89	0.51	147		5 150			√
**BS100**	9	D	0.14	213.4	1.86	1.72	162		4 860			√
**BS23-14**	9	D	0.159	69.1	1.82	1.84	90.8		2 720			√

Supplemental Table S9. Raw data metrics obtained from the different sequencing technologies used (Miseq, Nextseq550 & Mk1C)

Short-reads		***Miseq (one sample)***	***Nextseq550 (11 samples)***
Total Reads (bp)		11,123,081	122,415,649
PF Reads		8,293,843	-
Percentage of Reads Identified (%)		98.5734	-
[Min – Max] (%)		[98.5734 – 98.5734]	-
>= Q30		2.4G (89.1%)	36.7G (97.2%)
Raw Data Depth*		60x	83x
Long-reads		***Mk1C -1st***	***Mk1C – 2nd****
Run time		72h/72h	18h33mon/63h
Estimated bases		1.41 (Gb)	256.77 (Mb)
Reads generated		350.67 (k)	79.47 (k)
Estimated N50		6.51 (kb)	5.11 (kb)
Total data produced (pass/fail)		17.36 (GB)	3.14(GB)
Raw Data Depth*		20x	3.7x
Bases called	***Pass***	806.5 (Mb)	148.94 (Mb)
	Fail	324.25 (Mb)	54.21 (Mb)

**For average genome size equal to 40Mbp. Calculated per sample [(>=Q30/Average Genome Size)/Number of Samples]*

***The run was stopped, before 63h, following the failure of the flow cell.*

Supplemental Table S10. Summarize of the assembled genomes' quality control sequenced with Mk1C and Miseq

**Quality Check Tool**		*gfastats v1.3.6 (galaxy 0)*			*QUAST v5.2.0 (galaxy 1)*		
Sample / **Technology**		*BS6* / Mk1C	*BS6* / Mk1C	*BS23-14* / MiSeq	*BS6* / Mk1C	*BS6* / Mk1C	*BS23-14* / MiSeq
Base composition (A:C:G:T)	***A***	12,270,104	3,092,257	10,107,404	-		
	C	12,050,709	3,085,315	11,283,521			
	G	12,062,833	3,103,825	11,309,367			
	T	12,280,116	3,081,106	10,252,097			
GC content %		49.55	50.06	**52.60**	49.55	50.06	51.96
#scaffolds		1,034	768	95,214	-		
#contigs (>=0bp)		-			1,034	768	9,5214
#contigs (>= 1000bp)					1,024	758	4,212
Total scaffold length (>=0bp)		48,663,762	12,362,503	43,170,475	48,663,762	12,362,503	43,170,475
Total length (>= 1000bp)		-			48,656,619	12,354,841	29,122,079
#contigs (>= 1000bp)					1,033	768	7,736
Largest contig (bp)					1,332,670	71,686	138,133
Average scaffold length (bp)		47,036.60	16,097.01	453.40	-		
Scaffold N50 (bp)		204,514	21,489	6,829	204,514	21,489	**13,187**
Scaffold auN (bp)		336,269.41	24,540.13	13,596.34	**336,271.1**	24,540.1	**18,532.4**
Scaffold L50		57	193	**1,248**	57	193	637
Largest scaffold (bp)		1,332,670	71,686	138,133	-		
Smallest scaffold (bp)		246	541	78			
#gaps in scaffolds (bp)		0	0	2,203			
Total gap length in scaffolds (bp)		0	0	218,086			
Average gap length in scaffolds (bp)		0	0	99			
Gap N50 in scaffolds (bp)		0	0	100			
Gap auN in scaffolds (bp)		0	0	99.89			
Gap L50 in scaffolds		0	0	1,091			

Largest gap in scaffolds (bp)	0	0	100	
Smallest gap in scaffolds (bp)	0	0	10	
#paths	1,034	768	95,214	

Supplemental Table S11. Summarize of the eight assembled genomes' quality control based on gfstats/QUAST metrics for NextSeq550 outputs

gfastats or QUAST		*BS6*	*BS200*	*BS22-9*	*BS22-13*	*BS100*	*S3*	*GC9*	*BS23-14*
**Base composition (A:C:G:T)**	**A**	13,732,971	11,448,346	14,192,678	11,969,774	9,552,980	10,308,406	9,750,913	8,563,989
	**C**	13,422,307	13,527,335	**19,221,653**	16,181,013	9,384,618	9,556,120	8,784,988	9,658,467
	**G**	13,456,633	13,575,550	**19,272,835**	16,226,238	9,394,510	9,557,854	8,775,343	9,694,742
	**T**	13,812,472	11,542,001	14,282,653	12,087,590	9,601,119	10,317,125	9,757,726	8,666,971
**GC content %**	***gfstats***	**49.39**	54.10	**57.48**	57.39	49.51	48.10	47.37	**52.90**
	QUAST	49.32	**54.21**	**57.47**	57.39	**49.64**	**48.3**	**47.69**	52.14
**#scaffolds**		53,477	67,457	58,352	64,084	29,362	1,784	3,149	80,557
#contigs (>=0bp)		53 477	67 457	58 352	64 084	29 362	1 784	3 149	80 557
#contigs (>= 1000bp)		7 555	6 558	11 736	12 654	4 108	234	516	1 624
Total scaffold length (>=0bp)	***gfstats***	54,670,833	50,349,652	67,139,713	56,614,987	38,119,435	39,744,716	37,074,408	36,590,589
	QUAST	54,670,833	50,349,652	67,139,713	56,614,987	38,119,435	39,744,716	37,074,408	36,590,589
Total length (>= 1000bp)		42,705,255	38,628,939	**53,385,455**	40,455,650	30,408,403	39,502,054	36,642,697	29,209,248
#contigs (>= 1000bp)		14,120	11,504	19,819	22,603	8,386	275	652	2,296
Largest contig (bp)		196,398	109,089	66,855	98,120	795,057	**2,577,683**	977,231	347,930
Average scaffold length (bp)		1,022.32	746.40	1,150.60	883.45	1,298.26	**22,278.43**	11,773.39	454.22
Scaffold N50 (bp)	***gfstats***	6,975	7,059	5,147	2,575	11,878	911,563	223,650	37,419
	QUAST	9,547	9,806	6,270	3,415	15,909	911,563	224,581	54,525
Scaffold auN (bp)	***gfstats***	16,634.33	12,970.31	8,013.48	4,873.46	57,467.42	966,51.07	278,142.85	59,359.64
	QUAST	19 187	15 479	9085.2	5756.5	65 530	971 818	280 679	73 161
Scaffold L50	***gfstats***	1,559	1,597	3,219	5,021	565	15	49	225
	QUAST	1,104	1,102	2,500	3,476	392	15	48	150
Largest scaffold (bp)		196,398	109,089	66,855	98,120	795,057	2,577,683	977,231	347,930
Smallest scaffold (bp)		56	56	56	56	56	56	56	56
#gaps in scaffolds (bp)		2,469	2,602	1,739	1,561	1,873	79	154	93
Total gap length in scaffolds (bp)		246,450	256,420	169,894	150,372	186,208	5,211	5,438	6,420

Average gap length in scaffolds (bp)	99.82	98.55	97.7	96.33	99.42	65.96	35.31	69.03
Gap N50 in scaffolds (bp)	100	100	100	100	100	100	100	100
Gap auN in scaffolds (bp)	99.98	99.85	99.75	99.53	99.93	93.75	81.36	95.51
Gap L50 in scaffolds	1,233	1,283	850	752	932	27	28	33
Largest gap in scaffolds (bp)	100	100	101	109	100	100	100	100
Smallest gap in scaffolds (bp)	10	10	10	10	10	10	10	10
#paths	53,477	67,457	58,352	64,084	29,362	1,784	3,149	80,557

Supplemental Table S12. BandageInfo results of the different assembled genomes

Samples / Technology	*BS6 (1) / Mk1C*	*BS6 (2) / Mk1C*	*BS23-14 / Miseq*	*BS6 / NextSeq550*	*BS200 / NextSeq550*	*BS22-9 / NextSeq550*	*BS22-13 / NextSeq550*	*BS100 / NextSeq550*	*S3 / NextSeq550*	*GC9 / NextSeq550*	*BS23-14 / NextSeq550*
Node count	1,204	779	121,225	86,733	104,650	117,173	110,870	48,840	10,369	12,364	98,374
Edge count	1,166	122	165,273	116,499	141,069	157,054	147,147	65,165	13,904	14,934	131,747
Smallest edge overlap (bp)	0	0	77	55	55	55	55	55	55	55	55
Largest edge overlap (bp)	0	0	77	55	55	55	55	55	55	55	55
Total length (bp)	47,155,877	12,376,239	44,764,774	55,999,806	51,914,870	69,712,812	58,735,319	38,857,010	40,162,472	37,503,429	37,530,823
Total length no overlaps (bp)	47,155,877	12,376,239	35,481,346	51,233,451	46,175,730	63,273,302	52,676,739	36,181,425	39,599,987	36,888,254	32,316,548
Dead ends	1,228	1,468	3,010	272	829	**811**	3,073	599	630	3,068	7,366
Percentage dead ends	50.9967%	94.2234%	1.24149%	0.156803%	0.396082%	**0.346069%**	1.38586%	0.613227%	3.0379%	12.407%	3.74388%
Connected components	642	**740**	894	78	307	106	867	200	172	1,285	**3,588**
Largest component (bp)	40,430,379	**116,538**	40,273,361	55,971,088	51,743,831	**69,581,344**	58,001,190	38,605,504	28,471,710	12,829,353	36,515,644
Total length orphaned nodes (bp)	6143,085	11,816,562	917,454	26,246	169,001	106,440	350,486	98,191	7,055,155	19,355,498	10,03,516
N50 (bp)	**200,479**	21,456	2,459	2,770	2,804	1,506	1,073	4,365	10,5756	**29,666**	21,471
Shortest node (bp)	499	536	78	56	56	56	56	56	56	56	56
Lower quartile node (bp)	3,650	7,689	82	92	70	106	107	113	61	62	58
Median node (bp)	8,596	12,958	100	236	117	260	267	258	85	111	65

Upper quartile node (bp)	22,618	21,219	171	417	285	513	516	418	276	445	96
Longest node (bp)	**13,27,226**	71,686	48,201	118,480	65,102	34,440	94,477	385,822	**387,256**	258,647	262,184
Median depth	0	0	16.6392	25.8084	32.6659	15.3516	12.7189	21.5761	34.3652	73.8975	53.0873
Estimated sequence length (bp)	0	0	42,354,561	51,793,399	52,163,315	99,381,324	89,777,165	38,322,283	41,402,110	39,956,412	47,717,490

Supplemental Table S13. BUSCO results of the different assembled genomes

Sample / ***Tech***	*database*	*Complete (C)*	*Complete and single copy (S)*	*Complete and duplicated (D)*	*Fragmented (F)*	*Missing (M)*	*Number of scaffolds*	*Number of contigs*	*Total length (bp)*	*Percent gaps (%)*	*Scaffold N50 (Kb)*	*Contigs N50 (Kb)*
BS6 (1) / *Mk1C*	*Asco*	653	642	11	217	836	1,034	1,034	48,663,762	0	204	204
	Basi	**1,620**	1,607	13	35	109						
BS6 (2) / *Mk1C*	*Asco*	90	89	1	75	1,541	**768**	**768**	12,362,503	0	21	21
	Basi	**371**	371	0	57	1,336						
BS23-14 / *Miseq*	*Asco*	734	724	10	221	571	95,214	97,417	43,170,475	0.505	6	3
	Basi	**1,589**	1,580	9	83	92						
BS6 / *550*	*Asco*	612	598	14	267	827	53,477	55,946	54,670,833	0.451	6	4
	Basi	**1,377**	1,354	23	196	191						
BS200 / *550*	*Asco*	728	711	17	225	753	67,457	70,059	50,349,652	0.509	7	4
	Basi	**1,594**	1,575	19	80	90						
BS22-9 / *550*	*Asco*	544	486	58	325	837	58,352	60,091	**67,139,713**	0.253	5	4
	Basi	**1,261**	1,081	180	302	201						
BS22-13 / *550*	*Asco*	474	416	58	319	913	64,084	65,645	56,614,987	0.266	2	2
	Basi	**1,047**	907	140	394	323						
BS100 / *550*	*Asco*	677	671	6	244	785	29,362	31,235	38,119,435	0.488	11	5
	Basi	**1,491**	1,488	3	148	125						
S3 / *550*	***Asco***	**1,666**	1,661	5	5	35	1,784	1,863	39,744,716	**0.013**	**911**	**455**
	Basi	1,173	1,152	21	77	514						
GC9 / *550*	***Asco***	**1,665**	1,662	3	5	36	3,149	3,303	37,074,408	0.015	223	135
	Basi	1,162	1,149	13	91	511						
BS23-14 / *550*	*Asco*	791	783	8	196	719	80,557	80,650	36,590,589	0.018	37	35
	Basi	**1,709**	1,703	6	12	43						

Asco: the lineage dataset is *ascomycota_odb10* (*Creation date: 2024-01-08, number of genomes: 365, number of BUSCOs: 1706*)
Basi: the lineage dataset is *basidiomycota_odb10* (*Creation date: 2024-01-08, number of genomes: 133, number of BUSCOs: 1764*)

Supplemental Table S14. Cost and risk analysis between phenol-chloroform and our optimized protocol

**Parameter**	**Phenol-Chloroform Method**	**Optimized Protocol**
Reagent cost per simple	Low (but variable)	Moderate (fixed kit cost)
Hands-on time per 10 samples	High (>=120min)	Low (<90min)
Technical complexity	High (including phase separation, precipitation)	Low (all solutions are included in the kit)
Toxic reagents	Yes (phenol, chloroform)	No hazardous organic solvents
Health risk	High (corrosive, volatile, toxic)	Minimal
Waste disposal requirements	Hazardous chemical waste	Standard laboratory waste
Reproducibility	Operator-dependent	High (standardized reagents and well described steps at protocols.io: DOI: dx.doi.org/10.17504/protocols.io.8epv5r6yjg1b/
Suitability for routine multi-sample processing	Limited	High
